# Supplementary material for: Protocol for The Toxin Study: Understanding clinical and patient reported response of children and young people with cerebral palsy to intramuscular lower limb Botulinum neurotoxin-A injections, exploring all domains of the ICF. A pragmatic longitudinal observational study using a prospective one-group repeated measures design
Source: BMJ Open. 2021 Apr 21;11(4):e049542. doi: 10.1136/bmjopen-2021-049542 (PMC8061828; doi:10.1136/bmjopen-2021-049542)
Supplement: Author's manuscript [file bmjopen-2021-049542.draft_revisions.pdf]

BMJ Open is committed to open peer review. As part of this commitment we make the peer review history of every article we publish publicly available.

When an article is published we post the peer reviewers' comments and the authors' responses online. We also post the versions of the paper that were used during peer review. These are the versions that the peer review comments apply to.

The versions of the paper that follow are the versions that were submitted during the peer review process. They are not the versions of record or the final published versions. They should not be cited or distributed as the published version of this manuscript.

BMJ Open is an open access journal and the full, final, typeset and author-corrected version of record of the manuscript is available on our site with no access controls, subscription charges or pay-per-view fees (http://bmjopen.bmj.com).

If you have any questions on BMJ Open's open peer review process please email info.bmjopen@bmj.com

BMJ Open

Protocol for The Toxin Study: Understanding clinical and patient reported response of children and young people with cerebral palsy to intramuscular lower limb Botulinum Toxin-A injections, exploring all domains of the ICF: A pragmatic longitudinal observational study using a prospective one group repeated measures design

Journal:	*BMJ Open*
Manuscript ID	bmjopen-2021-049542
Article Type:	Protocol
Date Submitted by the Author:	28-Jan-2021

Complete List of Authors:	Katchburian, Lesley; Great Ormond Street Hospital For Children NHS Foundation Trust, The Wolfson Neurodisability Movement Disorder Service; UCL, GOSH Institute of Child<br>Oulton, Kate; Centre for Outcomes and Experience Research in Children's Health, Illness and Disability (ORCHID)<br>Main, Eleanor; University College London Institute of Child Health, Physiotherapy,<br>Morris, Christopher; University of Exeter Medical School,<br>Carr, Lucinda; Great Ormond Street Hospital for Children, Neuro Science

Keywords:	Paediatric neurology < NEUROLOGY, Developmental neurology & neurodisability < PAEDIATRICS, REHABILITATION MEDICINE

SCHOLARONE™
Manuscripts

I, the Submitting Author has the right to grant and does grant on behalf of all authors of the Work (as defined in the below author licence), an exclusive licence and/or a non-exclusive licence for contributions from authors who are: i) UK Crown employees; ii) where BMJ has agreed a CC-BY licence shall apply, and/or iii) in accordance with the terms applicable for US Federal Government officers or employees acting as part of their official duties; on a worldwide, perpetual, irrevocable, royalty-free basis to BMJ Publishing Group Ltd ("BMJ") its licensees and where the relevant Journal is co-owned by BMJ to the co-owners of the Journal, to publish the Work in this journal and any other BMJ products and to exploit all rights, as set out in our licence.

The Submitting Author accepts and understands that any supply made under these terms is made by BMJ to the Submitting Author unless you are acting as an employee on behalf of your employer or a postgraduate student of an affiliated institution which is paying any applicable article publishing charge ("APC") for Open Access articles. Where the Submitting Author wishes to make the Work available on an Open Access basis (and intends to pay the relevant APC), the terms of reuse of such Open Access shall be governed by a Creative Commons licence – details of these licences and which Creative Commons licence will apply to this Work are set out in our licence referred to above.

Other than as permitted in any relevant BMJ Author's Self Archiving Policies, I confirm this Work has not been accepted for publication elsewhere, is not being considered for publication elsewhere and does not duplicate material already published. I confirm all authors consent to publication of this Work and authorise the granting of this licence.

For peer review only

Protocol for The Toxin Study: Understanding clinical and patient reported response of children and young people with cerebral palsy to intramuscular lower limb Botulinum Toxin-A injections, exploring all domains of the ICF: A pragmatic longitudinal observational study using a prospective one group repeated measures design

Authors: Lesley Katchburian[1,3] lesley.katchburian@gosh.nhs.uk, Kate Oulton[2] kate.oulton@gosh.nhs.uk, Eleanor Main[3] e.main@ucl.ac.uk, Christopher Morris[4] Christopher.Morris@exeter.ac.uk, Lucinda Carr[1] lucinda.carr@gosh.nhs.uk,

Affiliations: [1] The Wolfson Neurodisability Movement Disorder Service, Great Ormond Street Hospital for Children NHS Foundation Trust, London, UK., [2] The Centre for Outcomes and Experience Research in Children's Health, Illness and Disability (ORCHID), Great Ormond Street Hospital for Children NHS Foundation Trust, London, UK., [3] Faculty of Population Health Sciences, University College London Great Ormond Street Institute of Child Health, London, UK. [4] Peninsula Childhood Disability Research Unit (PenCRU), University of Exeter Medical School, Exeter, UK.,

Corresponding Author: Lesley Katchburian, Lesley.katchburian@gosh.nhs.uk. The Wolfson Neurodisability Movement Disorder Service, Great Ormond Street Hospital for Children NHS Foundation Trust, London, UK.

Keywords: Cerebral palsy, Botulinum Toxin-A, hypertonia, ICF, Quality of movement

Word Count: 4229 words

ABSTRACT

Introduction: Botulinum Toxin-A (BoNT-A) is an accepted treatment modality for the management of hypertonia in children and young people with cerebral palsy (CYPwCP). Nevertheless, there are concerns about the long-term effects of BoNT-A, with a lack of consensus regarding the most meaningful outcome measures to guide its use. Most evidence to date is based on short-term outcomes, related to changes at impairment level (restrictions of body functions and structures), rather than changes in adaptive skills (enabling both activity and participation). The proposed study aims to evaluate clinical and patient reported outcomes in ambulant CYPwCP receiving lower limb BoNT-A injections over a 12-month period within all domains of the World Health Organisation's ICF and health-related quality of life (HRQoL).

Methods and Analysis: This pragmatic prospective longitudinal observational study will use a one group repeated measures design. Sixty CYPwCP classified as Gross Motor Classification Levels I-III aged between 4 and 18 years, will be recruited from an established motor disorders service in London, UK. Standardised clinical and patient reported outcome measures within all ICF domains; body structures and function, activity (including quality of movement), goal attainment, participation and HRQoL will be collected pre-injection and at 6 weeks, 6 months and up to 12 months post injection. A representative subgroup of children and carers will participate in a qualitative component of the study, exploring how their experience of BoNT-A treatment relates to clinical outcome measures.

Ethics and Dissemination: Central London Research Ethics Committee has granted ethics approval (#IRAS 211617 #REC 17/LO/0579). Findings will be disseminated in peer-reviewed publications,

For peer review only - http://bmjopen.bmj.com/site/about/guidelines.xhtml

conferences, and via networks to participants and relevant stakeholders using a variety of accessible formats including social media.

ARTICLE SUMMARY

Strengths and limitations of this study

1. To our knowledge this is the first published use of the 'Quality Function Measure' (QFM) to objectively evaluate quality of movement (QoM) following BoNT-A treatment and explore whether any change in QoM translates into changes in outcome within activities, participation and health related quality of life.

2. This pragmatic mixed methods observational study will evaluate response to lower limb botulinum A (BoNT-A) injections in ambulant CYPwCP using standardised outcome measures within ***all*** domains of the ICF over a twelve-month period.

3. This study is designed to optimise targeting of BoNT-A treatment by identifying patterns of response to BoNT-A treatment across all ICF domains in order to assist clinicians and families in making informed decisions about future treatment.

4. This is a pragmatic one group study design, conducted from a single tertiary (specialist children's) centre. Participants will continue to receive their routine rehabilitation therapy and orthotic provision at local community level. Individualized intervention parameters may make study replication difficult in other settings.

For peer review only

INTRODUCTION

Cerebral Palsy (CP) is the most common cause of physical disability in childhood (1). Although the initial brain insult is described as static, the effects of the neurological involvement are dynamic and change with time and growth of the child (2) Increased tone (hypertonia) is considered one of the primary motor impairments in children and young people with CP (CYPwCP)(3) and a significant contributor to secondary musculoskeletal impairments impacting on activity and participation(4).

Since the 1990's, intramuscular Botulinum neurotoxin A (BoNT-A) has become an internationally accepted treatment modality for the management of hypertonia in overactive muscle groups (5). BoNT-A once injected into the hypertonic muscle produces a 'reversible' temporary localised muscle weakness by blocking acetylcholine release at the neuromuscular junction. The pharmacological effect is said to last for 12-16 weeks, and the ability of BoNT-A to reduce focal hypertonia in ambulant CYPwCP has been well documented (6-8). Whilst the effects can be observed within 24-72 hours following injection, the period of clinically useful relaxation is reported to last between three to six months (9, 10).

The progression of dynamic contracture to fixed contracture is a fundamental issue in the care of the child with CP. The period of decreased hypertonia following BoNT-A provides a 'window of opportunity' for therapy to address specific predetermined goals of rehabilitation, such as stretching and strengthening of muscles, increased range of motion of joints, improved postural management and pain relief. Despite the temporary effect of injections, gains in motor function have been reported to last as long as twelve months (11).

Several studies have demonstrated the benefits of BoNT-A injections for ambulant CYPwCP in Gross Motor Functional Classification System (GMFCS) Levels I-III (12), particularly at single level use (injection at one level; e.g. the calf complex to treat equinus foot posture) (3, 13-17) . A meta-analysis of double-blind, randomized controlled clinical trials (RCT's) confirmed superiority of BoNT-A over placebo injections into the calf complex on improvement of gait in patients with spastic equinus (18). BoNT-A treatment, in combination with other rehabilitation treatments , has resulted in a significant improvement in functional goal attainment over and above those in a non-BoNT-A treatment group (19), with improvements in gait parameters, pain reduction and splint tolerance (20-23).

Nevertheless, despite positive outcomes for single level use, RCTs of the effectiveness of multi-level use of BoNT-A injections report mixed results. (24, 25). Although a recent systematic review of interventions for CYPwCP, identified BoNT-A treatment as one of few interventions with a sound evidence base (26). A recent Cochrane Collaboration report by Blumetti et al., reviewed 31 studies

For peer review only

assessing 1508 participants and concluded that there is limited evidence to show that compared to placebo or usual care BoNT-A improves walking, joint motion, satisfaction with outcome of treatment and muscle spasticity in CYPwCP (27). Sample sizes in BoNT-A studies are often small and predominately based on short-term outcome (3-6 months) with few assessing outcomes beyond six months.

Although it is widely acknowledged that BoNT-A treatment is not a 'stand-alone' treatment, detailed information regarding the adjunctive measures used in conjunction with BoNT-A is often lacking, making evaluation of its efficacy difficult (28-30). Some authors have highlighted the difficulty in relating changes in impairment measures following BoNT-A treatment to functional improvements in CYPwCP, with little reference made to minimal clinically important differences (MCIDs) (24). This raises concerns that current standardised outcome measures, focusing predominantly on impairment measures (without relating this to other domains of the ICF), may lack the sensitivity to pick up meaningful changes following injections, or indeed overestimate treatment effects if these do not relate to MCIDs, all highly pertinent when planning repeat treatment (24, 27, 31).

Recent studies investigating pathophysiological changes within hypertonic muscle have highlighted potential histological changes following both single and repeated BoNT-A treatment (32). A number of authors have suggested potential harm following repeated BoNT-A use (33-36). However, both positive and negative effects have been reported and a variability in measurement techniques and muscles assessed, makes comparison between studies challenging (37-42). Although BoNT-A has been described as a 'reversible treatment' (43), some authors suggest that BoNT-A exposure in CYPwCP may be associated with impaired muscle growth in the short term (44-47) and potential long term atrophy (41, 45, 48).

The WHO's International Classification of Functioning, Disability and Health (ICF) encourages evaluation of adaptive skills (enabling activities and participation) and health related quality of life (HRQoL) in order to target interventions that are meaningful to CYPwCP and their families (Figure 1). However evidence for BoNT-A treatment remains mostly related to measures of body functions and structures and less evidence pertains to activity and participation (22, 25, 49-51). Few trials have explored improvement in the activity and participation domains or QoL, after BoNT-A injections (24, 52) and qualitative data relating to CYP and caregivers experience is rarely incorporated (53-55).

Figure 1: World Health Organisation's ICF schematic representation of living with a health condition.

Longitudinal changes are not well characterised and evidence of impact on CYPwCP is elusive. The uncertainty that exists around BoNT-A treatment has resulted in a call to extend the period that CYPwCP are followed up after BoNT-A treatment beyond the short term, 12 week period (44), with

For peer review only - http://bmjopen.bmj.com/site/about/guidelines.xhtml

an imperative to evaluate interventions with BoNT-A using more sensitive outcome measures that evaluate meaningful aspects of health and quality of life for CYPwCP and families. (35, 44, 56-58)

Concern has been raised that BoNT-A treatment may be overprescribed for CYPwCP (35, 36, 38) if there is little guidance about which patients will benefit, risking potential harm if outcomes are unclear (38, 59-61). Whilst clinical evidence suggests that BoNT-A remains a valuable treatment option (15, 58, 62, 63) , there is a need to optimise it's use by developing clear guidelines and robust treatment algorithms in order to predict which children and young people will benefit from the addition of BoNT-A intervention to their overall management programme and when it is preferable to consider other treatment options.

The Toxin Study

This paper describes the protocol for a pragmatic prospective longitudinal observational study in an established paediatric movement disorder service in London, UK. As BoNT-A treatment is considered best practice care for focal hypertonia management in CP (19, 21, 48) there are practical and ethical concerns regarding the inclusion of a 'no treatment' control group and so a comparator was deemed not ethically appropriate.

The aims of this study are to:

1. Investigate clinical and patient reported outcomes (of body structures and function, quality of movement, activities and participation and health-related QoL) associated with lower limb BoNT-A injections in ambulatory CYPwCP over a twelve-month period.
2. Determine any factors associated with a response to BoNT-A treatment.
3. Explore qualitatively how standardised clinical outcome measures relate to the experiences of CYPwCP following BoNT-A treatment.

METHODS

Study design

This is a mixed methods study comprising of two phases:

Phase 1: To meet objectives 1 and 2 we will use a prospective longitudinal study using a one group repeated measures design with each child acting as their own control.

Phase 2: To meet objective 3 we will conduct interviews with a subgroup of CYPwCP and parent/carers from Phase 1 to elicit their experiences, and views of change following BoNT-A treatment.

For peer review only

Using a convergent mixed methods approach, quantitative data from Phase 1 will be synthesised with qualitative data from phase 2 in order to gain understanding of the impacts of BoNT-A treatment (55, 64).

Study sample and recruitment

Potential study participants will be identified from clinical lists of the Movement Disorder Service at Great Ormond Street Hospital for Children (GOSH). All eligible participants will be invited to take part in Phase 1 and will be enrolled sequentially. A subgroup of CYPwCP and their parents/primary carers will then be invited to participate in Phase 2 following review at six months post injection. Purposive sampling will be used to ensure a representative sample of CYPwCP within each GMFCS level.

Inclusion and exclusion criteria

Participants with a confirmed diagnosis of CP meeting the following criteria will be included:(1) ambulant, functioning at GMFCS level I-III; (2) aged between 4 and 18 years (3) prescribed lower limb BoNT-A injections in their clinical management plan for dynamic hypertonia limiting functional goals or causing pain.

Children will be excluded if they have previously had: (1) orthopaedic surgery to the injected muscle (2) neurosurgery for tone reduction (Selective Dorsal Rhizotomy), (3) lower limb BoNT-A injections in the last 6 months, or currently have (4) unrelated musculoskeletal problems such as recent acute injury or congenital structural abnormality, (5) no access to a block of therapy (defined as a minimum of six weekly sessions) post injections, (6) an inability to complete baseline assessments due to capacity, ability or willingness.

Parents: Every effort will be made to support the inclusion of all families invited to participate in the study (including those where English is not their first language). We will use translators when required to ensure that there is sufficient understanding to complete the measures.

INTERVENTION

Motor assessment

An experienced multidisciplinary team (consisting of a Consultant Paediatrician/ Neurologist and Senior Physiotherapist) will identify muscle groups to be injected. Muscle selection will vary between participants according to presence of dynamic hypertonia (based on clinical assessment with reference to standard definitions (65)), related functional impairment and individual goals of the

For peer review only

CYPwCP. All participants will receive a six-week block of therapy following injections from their local therapy teams, delivered locally in the community as per usual care. Details of dosage including frequency, location and type of therapy (e.g. goal directed therapy, strengthening) together with participation activities will be recorded including any additional intervention such as casting or change in orthotic provision. This will be required in order to describe the content and parameters of usual care in as much detail as possible (66).

Administration of BoNT-A

We will follow standard clinical practice prescription of BoNT-A at GOSH which involves administration of 500 U Dysport® (abobotulinumtoxinA, Dysport® Ipsen Ltd) diluted in 1 ml of normal saline, up to a maximum dose of 30 units /kg/body weight or a total dose up to a maximum of 1000 units per injection session. All CYPwCP will continue to receive BoNT-A injections under ultrasound guidance as a day case, either under sedation with local analgesia, or under general anaesthetic. Adverse events will be recorded and standard reporting of dose per muscle and follow up will be as per current clinical policy.

Training and fidelity

Clinical staff collecting study data are experienced members of the tertiary service with extensive experience of working with CYPwCP (mean 21 years, range 13-33 years). A standardised protocol for measurement and documentation is used in the clinic and an additional study manual with instructions for clinicians will be used to ensure consistency during the study. Two half day training sessions will be provided for clinicians collecting study data prior to the start of recruitment. Monthly meetings with clinicians by the research team will ensure consistency and adherence to study protocol.

Study Procedures & Data Collection

Staggered patient recruitment and data collection will commence in September 2017 and each participant will be followed up for a period of twelve months (**Error! Reference source not found.**). Data collection and final analysis of participant data is expected to be completed by September 2021. All decisions regarding clinical care, assessment frequency and BoNT-A injections will continue as per usual clinical practice. Standardised clinical assessments and outcome measures will be performed at four time points T_0 -T_3. The timings and rationale for these are summarised in Table 1

For peer review only

T_0	**Pre-injection baseline measures**	**1- 6 weeks before injection**
T_1	**6 weeks post injection**	*Estimated time to reach target threshold for BoNT- A* ***'evaluation of efficacy of injections'***
T_2	**6 months following injection**	*Expected completion of pharmacological action -* ***'evaluation of retention of effects post injection'***
	At T_2 **as per usual clinical practice** there are three possible outcomes for participants<br>• Favourable response to injections with retention of effects – no further injections indicated at this time<br>• Favourable response to injections- listed for a second injection cycle<br>• Non favourable response to injections- discharged to other services (e.g. neurosurgery/orthopaedics)	
T_3	**12 months following initial injection**	**End of study**

Table 1: Timings of study assessments T0-T3

All participants *who have not undergone a surgical procedure* will be assessed at T_3, independent of outcome at T_2. This will facilitate analysis of factors associated with changes in impairment, activity, participation and HRQoL following BoNT-A treatment and evaluate time to re-injection over 12 months. . The need for re-injection will be determined by clinical examination (documentation of a technical response e.g. change in MTS), evaluation of goal (COPM) scores and in consultation with families and local team as per usual clinical practice.

Figure 2: Study design flowchart of patient recruitment and data collection during the study

Validated outcome measures for all ICF domains

The standardised outcome measures used in the study are summarised within the ICF domains in Figure 3 with administration details of the measures summarised in Table 2. Outcome measures follow GOSH standard clinical practice with primary outcome measures marked in italics*. Patient assessment takes between 60-90 minutes.

Figure 3: Schematic representation of ICF Domains including standardised outcome measures used in the study.

For peer review only

Table 2: Summary of outcome measures used in the study

ICF Domains	Assessment	Outcome Measures	Method of Administration	Units	Description
Body functions and structures<br><br>**(impairment)**	Hypertonia and dynamic range of movement	**Modified Tardieu Scale (MTS)**	Standardised goniometry placement	Degrees	**MTS** measured at injected muscles. The difference between the slow stretch R_2 and a fast stretch R_1 is reported as the 'dynamic range' and when a difference is present this 'dynamic range' is reported to be amenable to treatment with BoNT-A.
	Pain	**Faces Pain Scale (FPS-R)**	Score assigned by CYP	Score 0- 10	**FPS-R** which has been shown to have good psychometric properties for pain reporting (modified for use by carer when CYP unable to self-report)
	Selective Motor Control	**Selective Motor Control scale (SMC)**	Score assigned by clinician	Score 0-4	**SMC** Selective motor control of the ankle is assessed using a standardised test procedure
Activity<br>**(functional limitation)**	Gross motor function	**Gross Motor Function Measure (GMFM) (D&E walk/run/jump dimensions)**	**Video recorded****<br>Standardised assessment form<br>Scored by clinician	% score	The **GMFM is** designed to evaluate change in gross motor function over time in CYPwCP . It is the standard outcome assessment tool for clinical intervention in CP Dimensions D& E will be used and are considered a **Proxy Functional Gait Measure**<br>*Families are consented for video storage in accordance with the Great Ormond Street Hospital policy*
	Quality of Gross Motor Function	***<u>Quality-Function Measure</u> (QFM)******	** scored by PI later from GMFM above	% score for 5 quality attributes	The ***QFM*** an observational validated measure captures the quality of movement of the GMFM (D&E) as above post BoNT-A may be more sensitive to change than GMFM alone <u>*scored from video no extra assessment time for CYP*</u>
	Balance/ functional mobility	**Modified Timed up and Go Test (mTUG)**	**Timed standardised test**<br>(from sitting CYP stands and walks distance 3m touches star returns to seat)	Seconds	**mTUG** integrates transitions and walking skills and provides a meaningful measure of capability. It has been shown to be a reliable outcome measure for assessing functional mobility in CYPwCP. **Proxy Functional Gait Measure**
	Walking ability (Efficiency)	**1-minute fast walk test (1MFWT)**	**Distance recorded**<br>(5 minutes rest followed by walking for **1** minute in a 9m corridor at maximum walking speed without running. CYP use normal walking aids and wear orthoses).	Metres	**1MFWT** is a good discriminator of functional ability for dynamic balance, muscle performance and endurance.<br>**Proxy Functional Gait Measure**
Participation<br>**(restriction)**	Involvement in daily activities	**Participation and Environment Measure for Children and Youth' (PEM-CY)**	**Parent- reported Questionnaire**<br>(25 item~ 25 minutes to complete)<br>*Answered at home or in clinic*	Summary Score	The **PEM-CY** assesses participation frequency and involvement in home school and community, along with environmental factors within these settings. Can be completed online at home or in clinic on a handheld device/ paper format whilst waiting for the child's assessment to be completed.
Health Related Quality of life<br>H	Quality of life across seven domains	**Cerebral Palsy Quality of life measure (CPQOL)**	**CYP (or proxy) reported Questionnaire**<br>CP QOL-Child / CP QOL Teen<br>*Answered at home or in clinic*	Mean Domain Score	The ***CPQOL-Child*** is designed to assess condition specific quality of life of children across seven domains for children aged 4-12 years - parent report for children aged 4 to 12 years and a self-report for children aged 9 to 12 years (52-66 items).<br>***CPQOL- Teen*** 13-18 years. Adolescent self-report version and a parent version (72-89 items).
Goal setting<br>**(ICF domains)**	Selection of goals CYP hope to improve following BoNT-A	***Modified Canadian Occupational Performance Measure (mCOPM)***	In clinic 3 Goals set by CYP and family pre injection<br>Scores assigned post injection	Score (1-10)	**mCOPM** rates perception and satisfaction of CYP's performance on a 1–10 ordinal scale. A score change of> two points is considered clinically significant

Classification of the participants

Gross Motor Function Classification System -Expanded and Revised (GMFCS-E&R)

The GMFCS-E&R (12) is an internationally recognised 5 level system to classify the motor abilities of CYPwCP aged 4-18 years, with Level I CYPwCP being identified as the most physically able and Level V the least. It is valid and reliable and frequently used in both research and clinical practice. Only ambulant CYPwCP classified as GMFCS Levels I-III will be included in this study.

Classification of Cerebral Palsy

Participants are classified according to the distribution (unilateral or bilateral) and tone presentation (hypertonia will be identified as predominantly spastic, dystonic or mixed in type) as identified by SCPE (67)

OUTCOME MEASURES

Primary outcome measures

Quality Function Measure QFM[1] is an observational criterion referenced measure designed to evaluate the Quality of Movement (QoM) in standing and walking skills in CYPwCP (68). It is used in conjunction with the Gross Motor Function Measure (GMFM) using Dimensions D and E, which focus on 'standing' and 'walking, jumping and running skills' which are considered proxy clinical gait measures The GMFM is the 'gold standard' tool for evaluating gross motor function in CYPwCP, evaluating 'how much' of a gross motor skill a child can perform (69). However, there are concerns regarding GMFM's sensitivity in capturing subtle yet meaningful change post intervention, due to 'ceiling effects' of the measure when used with ambulant CYPwCP in GMFCS levels I-III (22, 68, 70). The QFM scores movement quality and assesses 'how well' a child performs gross motor tasks (68). It has shown excellent rater and test-retest reliability (ICC 0.89-0.97). Minimal detectable change estimates (6-9%) suggest that the scale has potential as an evaluative measure (V.Wright, personal communication, May 8, 2018). However, to date, there are no published studies evaluating the responsiveness of QFM following BoNT-A injections.

In order to minimise bias, the video analyst will be blinded to the stage of treatment and assessment time point (i.e. pre T_0 or post $T_{1\text{-}3}$ injection). To conceal these time points, each video containing GMFM D & E (standing and walking) dimensions is anonymised and randomly allocated a letter by a co-worker not involved in the service. To minimise recall bias, a time lag will be built in of two weeks and videos of at least 10 other children will be scored before scoring the video of the same child at a

[1] Primary outcome measures for The Toxin Study

For peer review only

different assessment time point. This is in keeping with previous QFM reliability studies which suggest a gap of two weeks before evaluating test-retest scores (68, 70). Data will be entered into a secure data base without access to previous scores until scoring for all assessment time points is complete.

Canadian Occupational Performance Measure (COPM)[1] is a goal attainment tool modified for use in the paediatric population and frequently used in neurodisability research (71-73). It identifies concerns regarding 'occupational performance issues' (OPIs) i.e. the ability to carry out functional tasks allowing the identification of goals and has been used to document change post BoNT-A rehabilitation (74). In the paediatric population areas of concern in a child's self-care, activity and leisure are explored during the pre-assessment appointment with the clinical team. COPM has demonstrated high re-test reliability (ICC 0.76-0.89), sensitivity to change and good content, construct and criterion validity for CYPwCP receiving BoNT-A (75, 76). Families are asked to identify up to three areas of concern which they and their child hope to improve following lower limb BoNT-A injections. Whenever possible, goals are set with the child's input (77). The child and parents are asked to rate their perception of both current performance and their satisfaction with this performance on a 1–10 ordinal scale. A score change of two or more points is considered clinically significant (72, 78).

Secondary outcome measures

Modified Tardieu Scale (MTS) is a recognised clinical measure to differentiate dynamic spasticity from fixed contracture in a muscle. It determines the passive range of movement at two different movement velocities fast (R1) measuring dynamic muscle length and slow (R2) measuring static muscle length, with the relative difference between the two (R2-R1) determining the dynamic tone component of the muscle contracture (79, 80). It is measured in degrees with a universal goniometer using standardised testing positions for each muscle. It has been postulated that the larger the dynamic tone component, the more amenable it is to treatment with BoNT-A (78). It is more effective than the Modified Ashworth Scale (MAS) in identifying the presence of spasticity (88.9%, kappa= 0.73, p=0.000) and the presence of contracture (77.8%, Kappa=0.503, p=008).

Faces Pain scale (FPS-R) is a self-report measure of pain intensity developed for children which has been shown to have good psychometric properties for pain reporting (modified for use by carer when the child is unable to self-report). The scale shows a close linear relationship with visual

For peer review only

analogue scales. It is measured by an ordinal scale from 0-10 with pictorial representation of faces. A change score of 2 or more is said to be clinically significant (81).

Selective Motor Control (SMC) is a good discriminator of selective motor control and assesses a child's ability to voluntarily and selectively control the dorsiflexors of the ankle (77). It is measured by an ordinal scale between 0 and 4. Higher score indicates better selectivity, inter-rater agreement has been shown to be moderate to good (Kw= 0.58-0.77) with strong test-retest reliability (Kw=0.88-1) (79, 82).

One Minute Fast Walk Test (1MFWT) has been chosen as a proxy gait measure and is a good discriminator of functional ability for dynamic balance, muscle performance and endurance (83). Participants walk for 1 minute along a 9-metre corridor at maximum speed without running. They are able to use their usual walking aids and orthoses. Distance is calculated to the nearest 10cm. It shows concurrent validity with the GMFM, with significant correlation between GMFM score and distance walked (r=0.92) and good reliability (ICC: 0.97)(84).

Modified Timed Up and Go (mTUG) is a good discriminator of balance, anticipatory postural control, and functional mobility in CYPwCP. Participants are timed rising from a chair, walking 3 metres touching a star on the wall and returning to sit down as quickly as they can without running. Participants are able to use their usual walking aids and orthoses. The measure differentiates performance between CYPwCP at GMFCS levels I-III. It has shown excellent inter-rater reliability (ICC 0.83-0.99)(85)

Participation Environment Measure -Child and Youth (PEM-CY) is an innovative parent reported participation measure for use with CYP between 4-18 years. It measures CYPwCP's participation at home (10 items), school (5 items) and community (10 items), taking into account the environmental challenges within each setting. Summary scores for participation frequency, child's involvement and parental desire for change are calculated for all domains. PEM-CY has demonstrated good internal consistency (ICC 0.72-0.83) and test-retest reliability (ICC 0.76-0.89) (86).

Cerebral Palsy Quality of Life Measure (CPQOL) is a HRQoL assessment administered by questionnaire, specifically designed for CYPwCP. It quantifies 'well-being' across seven key HRQoL domains. Items are scored on a nine-point rating scale, then summed and averaged to generate seven domain scores. There are two child reported (CPQOL-Child: 9-12y and CPQOL-Teen:13-18y) and two proxy reported versions dependent on child's age and cognitive ability (CPQOL-Child

For peer review only

Primary care giver 4-12 y and CPQOL-Teen Primary care giver13-18y). The questionnaire designers have stipulated a minimum age of 9 years for child self- reporting, however significant disagreement has been reported between child and parent proxy reports in many HRQoL instruments and the child's perspective will be sought whenever possible in this study (87). The CPQOL has demonstrated good internal consistency (Child ICC 0.74-0.92; Teen 0.81-0.96), and adequate test-retest reliability (Child ICC 0.76-0.89; Teen 0.59-0.83) (88, 89).

Semi-structured interviews

Interviews will be used to elicit CYPwCP and parent/carers views of change following BoNT-A treatment. These are expected to last 60 minutes for parents and up to 30 minutes for children and will take place at home or in the hospital (depending on family preference). Interviews will be guided by a predetermined interview schedule, which will explore perceived change across all ICF domains to facilitate comparison with quantitative outcome measures, as well as investigate the acceptability of the standardised outcome measures used in Part 1 of the study. Parents will be interviewed separately from CYPwCP although individual preference for parents to be present or absent during interviews with their child will be respected. Interviews with CYPwCP will be tailored to their age, cognitive and communication ability using a variety of different techniques, including art-based activities. Offering a toolkit of different creative techniques will ensure that the activities will be accessible to all CYPwCP involved in the interviews, acknowledging not only their different skills and personalities but also their cognitive and physical abilities which is particularly important in this population of CYP (90). To ensure a representative sample of participants for Part 2, CYPwCP with a good, moderate and poor response to toxin (determined by the clinical team at the six-month assessment) within each GMFCS level (I-III) will be invited to take part in Part 2. All interviews will be audio recorded with permission from participants.

Sample size

Phase 1: This study has been powered to detect a difference on one of the two primary outcome measures, The Canadian Occupational Performance Measure (COPM). The sample size power calculation is based on anticipated change in the COPM goal performance at the primary end point T1 (6 weeks post intervention) after BoNT-A treatment. A change in score of two or more points on the performance scale of the COPM would be considered clinically meaningful (72, 78). An earlier study of lower limb BoNT-A intramuscular injections yielded standard deviations between 1.4 and 1.7 for COPM performance (77) . Based on a conservative estimate using a mean change of two points on the COPM performance scale (power 0.8, two tailed, $p<0.05$), 36 participants (12 in each

For peer review only

GMFCS level) are required. Allowing for attrition and missing data over a twelve-month period a total of 60 participants (20 in each GMFCS level) will be recruited for Part I of the study.

As there are no studies reporting Quality Function Measure (QFM) (68) as a primary outcome measure following BoNT-A injections, no data exists to inform a power calculation. It is anticipated that the results from this study will provide power calculation data for use in future multi-centre trials using QFM.

Phase 2: A sample size of approximately 15 CYPwCP (5 from each of the GMFCS levels I-III) and their parent/carer is anticipated to be sufficient to reach thematic saturation for the qualitative element of the study (91)

Data Analysis

Descriptive analysis of the prospective study will provide information on baseline characteristics of impairment, activity, participation and HRQoL. Means and standard deviations will be used for normally distributed data and medians and inter-quartiles ranges for skewed data. Other demographic data will be described in a similar way with frequencies and proportions used for categorical information. The primary outcomes of the intervention will be assessed using generalised estimating equations for longitudinal analysis to evaluate differences in continuous data at post intervention time points. Multilevel regression models will be used to investigate the importance of the various potential predictors over a 12-month period on the 4 main outcomes, impairment, activity, participation and HRQoL Firstly, univariate relationships will be explored and then if appropriate we will fit a multivariable model for each outcome.

All interviews will be transcribed verbatim and transcripts checked by the interviewer. Qualitative data will be analysed using Braun and Clarke's six phase framework for thematic analysis Figure 4 (92). Two researchers (LK,KO) will undertake analysis of the transcripts to determine consistent themes and will then map these onto results from standardised outcomes. This will allow themes to emerge which can then be used to evaluate how closely standardised outcome measures relate to family experience.

Figure 4: Braun and Clarke's Six step approach to thematic analysis

Participant and Data management

Electronic data will be managed through a secure data base held in GOSH. Participants will be allocated an identification code which will be used to de-identify their files and forms. Pseudonyms will be used in all reports and publications and direct quotations will be anonymised. Paper documents and other manual files will be appropriately filed and stored securely in a locked filing

For peer review only

cabinet at GOSH. Demographic information and consent forms will be stored separately to research data. Classification measures, child demographics and related information will be taken prior to baseline for the purposes of stratification and description of the sample. Audio and Visual recordings will be uploaded onto secure password protected encrypted NHS computers and deleted from the recording device immediately after uploading. This protocol has been reviewed and approved by the Research and Development team and Caldecott Guardian at GOSH.

Patient and Public Involvement (PPI)

Children and young people with CP and their parents have been involved in the development of this project, exploring the importance of the research, the appropriateness of the research questions, the acceptability of the research methods and best methods for disseminating findings. Fifteen ambulant CYPwCP receiving BoNT-A treatment at GOSH and their parents were consulted individually. A wider population of CYPwCP and their parents were also consulted via the SCOPE website and the advisory groups for CYPwCP at Brunel University and Young People's Advisory Group (YPAG) at GOSH.

Reference Group/ Steering Group

Three parents (all mothers) and three CYPwCP (two boys and one girl-not participants) continue advising on the study through membership of the study steering group (parents) or reference group (CYP). Three professionals (two physiotherapists and one doctor) are also part of the steering group. By including CYPwCP and families perspectives together with regular contributions from practicing clinicians within the service we hope to ensure the inclusion of important values and preferences from families and clinicians alike, ensuring that the findings of the study remain relevant and applicable to the management of ambulant CYPwCP (24, 93, 94)

DISCUSSION

This paper presents the protocol for a novel pragmatic prospective longitudinal observational study of BoNT-A use in ambulant CYPwCP at an established tertiary children's centre in the UK. It will measure outcomes across all domains of the ICF and HRQoL over a 12-month period, to observe change and examine factors associated with positive or negative response to lower limb BoNT-A injections. The introduction of a standardised outcome measure, QFM, to evaluate any change in movement quality following BoNT-A treatment will further inform exploration of the relationship between tone reduction and changes in activity, as well as any influence on participation and HRQoL for our cohort of CYPwCP. . Mixed methods research designs are considered by many to be essential

For peer review only

in the study of therapeutic interventions in CP (55). Including qualitative study data will ensure that the experiences of CYPwCP following BoNT-A injections are elucidated, considered and further understood.

This study, by identifying patterns of response to BoNT-A injections in key aspects of health across all the ICF domains, will provide clinicians and families with meaningful information to inform future treatment planning and optimise the use of BoNT-A in CYPwCP. Evidence will be generated about the appropriateness of the outcome measures used in detecting meaningful change after BoNT-A injections as well as their acceptability to CYP. In addition, the relationship between the standardised outcome measures used to capture treatment effect and the perception of outcome by children and their families will be investigated.

The results of this study will assist in the development of a pragmatic set of standardised clinical outcome measures (recognising minimum clinically important differences) in order to evaluate the effects of BoNT-A treatment in this cohort of patients. We hope to inform future evaluative research; working towards closer consensus on patient selection, injection frequency and consideration of how long treatment should continue, in order to assess the value of long-term use of BoNT-A treatment and its role in the management of CYPwCP.

A dissemination strategy has been devised to ensure the findings of this research are made widely accessible to families and professionals in order to maximise impact on the care of CYPwCP. Working in partnership with parent groups will strengthen our dissemination strategy, ensuring findings are shared in a variety of accessible formats reaching a wide range of families, professionals, as well as academics and policy makers helping ensure the findings are translated into practice. We will disseminate the results of the study through international peer reviewed journals and at national and international conferences. A social media strategy will also be developed to ensure dissemination of a plain language summary of findings.

For peer review only

References

1. Odding E, Roebroeck ME, Stam HJ. The epidemiology of cerebral palsy: incidence, impairments and risk factors. Disabil Rehabil. 2006;28(4):183-91.
2. Molenaers G, Van Campenhout A, Fagard K, et al. The use of botulinum toxin A in children with cerebral palsy, with a focus on the lower limb. J Child Orthop. 2010;4(3):183-95.
3. Koman LA, Mooney JF, 3rd, Smith BP, et al. Management of spasticity in cerebral palsy with botulinum-A toxin: report of a preliminary, randomized, double-blind trial. Journal of Pediatric Orthopedics. 1994;14(3):299-303.
4. Koman LA, Paterson Smith B, Balkrishnan R. Spasticity associated with cerebral palsy in children: guidelines for the use of botulinum A toxin. Paediatric Drugs. 2003;5(1):11-23.
5. Ward AB, Molenaers G, Colosimo C, et al. Clinical value of botulinum toxin in neurological indications. European Journal of Neurology. 2006;13 Suppl 4:20-6.
6. Tedroff K, Granath F, Forssberg H, et al. Long-term effects of botulinum toxin A in children with cerebral palsy. Developmental Medicine & Child Neurology. 2009;51(2):120-7.
7. Hoare BJ, Imms C. Upper-limb injection of botulinum toxin-A in children with cerebral palsy: a critical review of the literature and clinical implications for occupational therapists. American Journal of Occupational Therapy. 2004;58(4):389-97.
8. Lukban MB, Rosales RL, Dressler D. Effectiveness of botulinum toxin A for upper and lower limb spasticity in children with cerebral palsy: A summary of evidence. Journal of Neural Transmission. 2009;116(3):319-31.
9. Ryll U, Bastiaenen C, Bie R, et al. Effects of leg muscle botulinum toxin A injections on walking in children with spasticity-related cerebral palsy: a systematic review (Provisional abstract). Developmental Medicine and Child Neurology. 2011;53(3):210-6.
10. Aoki KR, Ranoux D, Wissel J. Using translational medicine to understand clinical differences between botulinum toxin formulations. Eur J Neurol. 2006;13 Suppl 4:10-9.
11. Eliege de Souza MNACD, Renata Calhes Franco MFMS, Fabiano Politti CSO. Effect of Botulinum Toxin a on Spasticity and Function in Children with Cerebral Palsy: A Systematic Review. Med Sci Tech, 2014; 55: 11-15. 2014.
12. Palisano R, Rosenbaum P, Walter S, et al. Development and reliability of a system to classify gross motor function in children with cerebral palsy. Dev Med Child Neurol. 1997;39(4):214-23.
13. Desloovere K. A randomized study of combined botulinum toxin type A and casting in the ambulant child with cerebral palsy using objective outcome measures. European Journal of Neurology. 2001;8(SUPPL. 5):75-87.
14. Gough M, Fairhurst C, Shortland AP. Botulinum toxin and cerebral palsy: time for reflection? Developmental Medicine & Child Neurology. 2005;47(10):709-12.
15. Svehlik M, Kraus T, Steinwender G, et al. Repeated multilevel botulinum toxin A treatment maintains long-term walking ability in children with cerebral palsy. Ceska a Slovenska Neurologie a Neurochirurgie. 2012;75(6):737-41.
16. Delgado MR. The use of botulinum toxin type A in children with cerebral palsy: a retrospective study. European Journal of Neurology. 1999;6:S11-S8.
17. Delgado M, Tilton A, Gormley M, et al. Abobotulinumtoxina injection in muscles outside the gastrocnemius-soleus complex in pediatric patients with lower limb spasticity. Developmental Medicine and Child Neurology. 2017;59 (Supplement 3):80-1.
18. Cardoso ES, Rodrigues BM, Barroso M, et al. Botulinum toxin type A for the treatment of the spastic equinus foot in cerebral palsy. Pediatr Neurol. 2006;34(2):106-9.
19. Wallen M, O'Flaherty SJ, Waugh MCA. Functional outcomes of intramuscular botulinum toxin type A and occupational therapy in the upper limbs of children with cerebral palsy: A randomized controlled trial. Archives of Physical Medicine and Rehabilitation. 2007;88(1):1-10.
20. Heinen F, Desloovere K, Schroeder AS, et al. The updated European Consensus 2009 on the use of Botulinum toxin for children with cerebral palsy. European Journal of Paediatric Neurology. 2010;14(1):45-66.

21. Fehlings D, Novak I, Berweck S, et al. Botulinum toxin assessment, intervention and follow-up for paediatric upper limb hypertonicity: International consensus statement. European Journal of Neurology. 2010;17(SUPPL. 2):38-56.
22. Love SC, Novak I, Kentish M, et al. Botulinum toxin assessment, intervention and after-care for lower limb spasticity in children with cerebral palsy: International consensus statement. European Journal of Neurology. 2010;17(SUPPL. 2):9-37.
23. Metaxiotis D, Siebel A, Doederlein L. Repeated botulinum toxin A injections in the treatment of spastic equinus foot. Clinical Orthopaedics & Related Research. 2002(394):177-85.
24. Wright FV, Rosenbaum PL, Goldsmith CH, et al. How do changes in body functions and structures, activity, and participation relate in children with cerebral palsy? Developmental Medicine & Child Neurology. 2008;50(4):283-9.
25. Strobl W, Theologis T, Brunner R, et al. Best clinical practice in botulinum toxin treatment for children with cerebral palsy. Toxins. 2015;7(5):1629-48.
26. Novak I, McIntyre S, Morgan C, et al. A systematic review of interventions for children with cerebral palsy: state of the evidence. Developmental Medicine & Child Neurology. 2013;55(10):885-910.
27. Blumetti FC, Belloti JC, Tamaoki MJS, et al. Botulinum toxin type A in the treatment of lower limb spasticity in children with cerebral palsy. Cochrane Database of Systematic Reviews. 2019(10).
28. Williams S, Elliott C, Gubbay A, et al. The immediate effect of Botulinum Toxin Type-A on muscle morphology and strength in children with cerebral palsy. Developmental Medicine and Child Neurology. 2012;54:35-6.
29. Schasfoort FC, Pangalila RF, Dallmeijer AJ, et al. Evaluation of the added value of botulinum toxin treatment and intensive physiotherapy to improve body function and structure in children with spastic cerebral palsy. Developmental Medicine and Child Neurology. 2017;59 (Supplement 2):21.
30. Mathevon L, Bonan I, Barnais JL, et al. Adjunct therapies to improve outcomes after botulinum toxin injection in children: A systematic review. Ann Phys Rehabil Med. 2019;62(4):283-90.
31. Williams SA, Reid S, Valentine J, et al. Activity, participation and quality of life in children with cerebral palsy: Can a combined approach of strength training and botulinum neurotoxin type-A help? Developmental Medicine and Child Neurology. 2014;56:76.
32. Booth CM, Cortina-Borja MJ, Theologis TN. Collagen accumulation in muscles of children with cerebral palsy and correlation with severity of spasticity. Dev Med Child Neurol. 2001;43(5):314-20.
33. Hastings-Ison T, Thomason P, Sangeux M, et al. Botox injections for spastic equinus in ambulant children with cerebral palsy: A prospective 3D kinematic study. Developmental Medicine and Child Neurology. 2018;60 (Supplement 1):56-7.
34. Copeland L, Edwards P, Thorley M, et al. Botulinum toxin A for nonambulatory children with cerebral palsy: a double blind randomized controlled trial. The Journal of pediatrics. 2014;165(1):140-6.e4.
35. Multani I, Manji J, Hastings-Ison T, et al. Botulinum Toxin in the Management of Children with Cerebral Palsy. Paediatr Drugs. 2019;21(4):261-81.
36. Gough M. Does botulinum toxin prevent or promote deformity in children with cerebral palsy? Developmental Medicine & Child Neurology. 2009;51(2):89-90.
37. Tedroff K. Short and long term effects of Botulinum toxin A in children. Developmental Medicine and Child Neurology. 2009;51:10.
38. Sim E, Park E, Jung S. Architectural change of gastrocnemius muscle after botulinum toxin injection in children with cerebral palsy. Developmental Medicine and Child Neurology. 2012;54:20-1.
39. Legerlotz K, Matthews KG, McMahon CD, et al. Botulinum toxin-induced paralysis leads to slower myosin heavy chain isoform composition and reduced titin content in juvenile rat gastrocnemius muscle. Muscle & Nerve. 2009;39(4):472-9.

For peer review only

40. Eek MN, Pahlman M, Himmelmann K, et al. Muscle strength after botulinum neurotoxin injection in children with cerebral palsy. Developmental Medicine and Child Neurology. 2014;56:61-2.
41. Fortuna R, Horisberger M, Vaz MA, et al. Do skeletal muscle properties recover following repeat onabotulinum toxin A injections? Journal of Biomechanics. 2013;46(14):2426-33.
42. Minamoto VB, Suzuki KP, Bremner SN, et al. Dramatic changes in muscle contractile and structural properties after 2 botulinum toxin injections. Muscle & Nerve. 2015;52(4):649-57.
43. Ney JP, Joseph KR. Neurologic uses of botulinum neurotoxin type A. Neuropsychiatric disease and treatment. 2007;3(6):785-98.
44. Alexander C, Elliott C, Valentine J, et al. Muscle volume alterations after first botulinum neurotoxin A treatment in children with cerebral palsy: a 6-month prospective cohort study. Developmental Medicine & Child Neurology. 2018;60(11):1165-71.
45. Van Campenhout A, Verhaegen A, Pans S, et al. Botulinum toxin type A injections in the psoas muscle of children with cerebral palsy: Muscle atrophy after motor end plate-targeted injections. Research in Developmental Disabilities. 2013;34(3):1052-8.
46. Park ES, Sim E, Rha DW, et al. Architectural changes of the gastrocnemius muscle after botulinum toxin type A injection in children with cerebral palsy. Yonsei Medical Journal. 2014;55(5):1406-12.
47. Williams SA, Reid S, Elliott C, et al. Muscle volume alterations in spastic muscles immediately following botulinum toxin type-A treatment in children with cerebral palsy. Developmental Medicine and Child Neurology. 2013;55(9):813-20.
48. Barber L, Hastings-Ison T, Baker R, et al. The effects of botulinum toxin injection frequency on calf muscle growth in young children with spastic cerebral palsy: a 12-month prospective study. Journal of children's orthopaedics. 2013;7(5):425-33.
49. NICE. Spasticity in children and young people with non progressive brain disorders. Clinical Guideline 145; . London: National Institute for Health and Clinical Excellence. ; 2012.
50. Anne Whelan M, Delgado Frcpc Faan MR. Practice parameter: pharmacologic treatment of spasticity in children and adolescents with cerebral palsy (an evidence-based review): report of the quality standards subcommittee of the american academy of neurology and the practice committee of the child neurology society...Neurology. 2010 Jan 26;74(4):336-43. Neurology. 2010;75(7):669-.
51. Carr LJ, Cosgrove AP, Gringras P, et al. Position paper on the use of botulinum toxin in cerebral palsy. UK Botulinum Toxin and Cerebral Palsy Working Party. Archives of Disease in Childhood. 1998;79(3):271-3.
52. Read FA, Boyd RN, Barber LA. Longitudinal assessment of gait quality in children with bilateral cerebral palsy following repeated lower limb intramuscular Botulinum toxin-A injections. Research in Developmental Disabilities. 2017;68:35-41.
53. Lowing K, Thews K, Haglund-Akerlind Y, et al. Effects of Botulinum Toxin-A and Goal-Directed Physiotherapy in Children with Cerebral Palsy GMFCS Levels I & II. Physical & Occupational Therapy in Pediatrics. 2017;37(3):268-82.
54. Tilton AH. Evidence-based review of safety and efficacy of botulinum neurotoxin in cerebral palsy. Toxicon. 2015;93:S59.
55. Rosenbaum P. How do we know if interventions in developmental disability are effective? Developmental Medicine & Child Neurology. 2020;62(12):1344-.
56. Nguyen L, Mesterman R, Gorter JW. Development of an inventory of goals using the International Classification of Functioning, Disability and Health in a population of non-ambulatory children and adolescents with cerebral palsy treated with botulinum toxin A. BMC Pediatrics. 2018;18(1):1.
57. Hastings-Ison T, Blackburn C, Graham HK, et al. Optimum frequency of botulinum toxin injections to the gastrocsoleus in children with cerebral palsy-a randomised controlled trial. Developmental Medicine and Child Neurology. 2014;56:27.

For peer review only

58. Langdon K, Copeland L, Edwards P, et al. Comment on: "Botulinum Toxin in the Management of Children with Cerebral Palsy". Pediatric Drugs. 2019.
59. Hastings-Ison T, Sangeux M, Graham H. Botulinum Toxin A injections for spastic equinus in ambulant children with cerebral palsy: Longterm follow-up and time to surgery. Developmental Medicine and Child Neurology. 2017;59 (Supplement 3):33-4.
60. Multani I, Manji J, Tang MJ, et al. Sarcopenia, Cerebral Palsy, and Botulinum Toxin Type A. JBJS reviews. 2019;7(8):e4.
61. Montastruc J, Marque P, Moulis F, et al. Adverse drug reactions of botulinum neurotoxin type A in children with cerebral palsy: a pharmaco-epidemiological study in VigiBase. Dev Med Child Neurol. 2017;59(3):329-34.
62. Delgado M, Tilton A, Gormley M, et al. Dysport (abobotulinumtoxinA) injection in muscles in pediatric patients with lower limb spasticity. Neurology Conference: 69th American Academy of Neurology Annual Meeting, AAN. 2017;88(16 Supplement 1).
63. Dursun N, Bonikowski M, Dabrowski E, et al. Efficacy of Repeat AbobotulinumtoxinA (Dysport®) Injections in Improving Gait in Children with Spastic Cerebral Palsy. Developmental Neurorehabilitation. 2019:1-7.
64. Fetters MD, Curry LA, Creswell JW. Achieving integration in mixed methods designs-principles and practices. Health Serv Res. 2013;48(6 Pt 2):2134-56.
65. Graham HK, Rosenbaum P, Paneth N, et al. Cerebral palsy. Nature Reviews Disease Primers. 2016;2(1):15082.
66. Hoffmann TC, Glasziou PP, Boutron I, et al. Better reporting of interventions: template for intervention description and replication (TIDieR) checklist and guide. BMJ : British Medical Journal. 2014;348:g1687.
67. Gainsborough M, Surman G, Maestri G, et al. Validity and reliability of the guidelines of the surveillance of cerebral palsy in Europe for the classification of cerebral palsy. Dev Med Child Neurol. 2008;50(11):828-31.
68. Wright FV, Rosenbaum P, Fehlings D, et al. The Quality Function Measure: reliability and discriminant validity of a new measure of quality of gross motor movement in ambulatory children with cerebral palsy. Dev Med Child Neurol. 2014;56(8):770-8.
69. Boyce W, Gowland C, Rosenbaum P, et al. The Gross Motor Performance Measure: Validity and Responsiveness of a Measure of Quality of Movement1995. 603-13 p.
70. Tustin K, Gimeno H, Morton E, et al. Rater reliability and scoring duration of the Quality Function Measure in ambulant children with hyperkinetic movement disorders. Dev Med Child Neurol. 2016;58(8):822-8.
71. Law M, Baptiste S, Mills J. Client-centred practice: what does it mean and does it make a difference? Can J Occup Ther. 1995;62(5):250-7.
72. McColl MA, Law M, Baptiste S, et al. Targeted Applications of the Canadian Occupational Performance Measure. Canadian Journal of Occupational Therapy. 2005;72(5):298-300.
73. Kang M, Smith E, Goldsmith CH, et al. Documenting change with the Canadian Occupational Performance Measure for children with cerebral palsy. Developmental Medicine and Child Neurology. 2020;62(10):1154-60.
74. O'Neil ME, Fragala MA, Dumas HM. Physical therapy intervention for children with cerebral palsy who receive botulinum toxin a injections. Pediatr Phys Ther. 2003;15(4):204-15.
75. Fragala MA, O'Neil M E, Russo KJ, et al. Impairment, disability, and satisfaction outcomes after lower-extremity botulinum toxin a injections for children with cerebral palsy. Pediatr Phys Ther. 2002;14(3):132-44.
76. Cusick A, McIntyre S, Novak I, et al. A comparison of goal attainment scaling and the Canadian Occupational Performance Measure for paediatric rehabilitation research. Pediatr Rehabil. 2006;9(2):149-57.
77. Bjornson K, Hays R, Graubert C, et al. Botulinum toxin for spasticity in children with cerebral palsy: a comprehensive evaluation. Pediatrics. 2007;120(1):49-58.

For peer review only

78. Law M, Baptiste S, Carswell A, et al. Canadian Occupational Performance Measure. Ottowa, Ontario: COAT publications; 1998 1998//.
79. Boyd RN, Graham HK. Objective measurement of clinical findings in the use of botulinum toxin type A for the management of children with cerebral palsy. European Journal of Neurology. 1999;6:S23-S35.
80. Love SC, Valentine JP, Blair EM, et al. The effect of botulinum toxin type A on the functional ability of the child with spastic hemiplegia a randomized controlled trial. European Journal of Neurology. 2001;8 Suppl 5:50-8.
81. Hicks CL, von Baeyer CL, Spafford PA, et al. The Faces Pain Scale-Revised: toward a common metric in pediatric pain measurement. Pain. 2001;93(2):173-83.
82. Lowing K, Bexelius A, Carlberg EB. Goal-directed functional therapy: A longitudinal study on gross motor function in children with cerebral palsy. Disability and Rehabilitation. 2010;32(11):908-16.
83. McDowell BC, Kerr C, Parkes J, et al. Validity of a 1 minute walk test for children with cerebral palsy. Dev Med Child Neurol. 2005;47.
84. Scholtes VA, Becher JG, Janssen-Potten YJ, et al. Effectiveness of functional progressive resistance exercise training on walking ability in children with cerebral palsy: a randomized controlled trial. Res Dev Disabil. 2012;33.
85. Carey H, Martin K, Combs-Miller S, et al. Reliability and Responsiveness of the Timed Up and Go Test in Children With Cerebral Palsy. Pediatric Physical Therapy. 2016;28(4):401-8.
86. Coster W, Bedell G, Law M, et al. Psychometric evaluation of the Participation and Environment Measure for Children and Youth. Dev Med Child Neurol. 2011;53(11):1030-7.
87. Upton P, Lawford J, Eiser C. Parent–child agreement across child health-related quality of life instruments: a review of the literature. Quality of Life Research. 2008;17(6):895.
88. Davis E, Mackinnon A, Davern M, et al. Description and psychometric properties of the CP QOL-Teen: a quality of life questionnaire for adolescents with cerebral palsy. Res Dev Disabil. 2013;34.
89. Waters E, Davis E, Mackinnon A, et al. Psychometric properties of the quality of life questionnaire for children with CP. Developmental Medicine & Child Neurology. 2007;49(1):49-55.
90. O'Grady MG, Dusing SC. Reliability and Validity of Play-Based Assessments of Motor and Cognitive Skills for Infants and Young Children: A Systematic Review. Physical Therapy. 2015;95(1):25-38.
91. Fugard AJB, Potts HWW. Supporting thinking on sample sizes for thematic analyses: a quantitative tool. International Journal of Social Research Methodology. 2015;18(6):669-84.
92. Braun V, Clarke V. Using thematic analysis in psychology. Qualitative Research in Psychology. 2006;3(2):77-101.
93. INVOLVE. Involving children and young people in research: Top tips and essential key issues for researchers. INVOLVE, Eastleigh 2016.
94. Nguyen L, Di Rezze B, Mesterman R, et al. Effects of Botulinum Toxin Treatment in Nonambulatory Children and Adolescents With Cerebral Palsy: Understanding Parents' Perspectives. Journal of Child Neurology. 2018;33(11):724-33.

Figure Legends

Figure 1: World Health Organisation's ICF schematic representation of living with a health condition 4
Figure 2: Study design flowchart of patient recruitment and data collection during the study 8
Figure 3: Schematic representation of ICF Domains including standardised outcome measures used in the study 8

For peer review only

Figure 4: Braun and Clarke's Six step approach to thematic analysis...14

Authors contributions

LK, KO, LC, EM, CM conceived and designed the study. LK developed the protocol with input from all authors. LK and LC are responsible for recruiting and supporting all the participants. LK, EM, KO will conduct the data analyses. LK and LC wrote the first draft of the manuscript, all authors reviewed this and have approved the final manuscript.

Acknowledgements

With thanks to the clinical team at The Wolfson Neurodisability Movement Disorder Service at GOSH, especially, Dr. Belinda Crowe, Xanthe Hodgson, Josephine Scerri, Emilie Hupin, Katharine Phillipps, Tessa Burnett, Sarah Foster, Agnieszka Rozborska, Stephanie Cawker. Also Dr Jo Wray from the Centre for Outcomes and Experience Research in Children's Health, Illness and Disability (ORCHID) at GOSH for support and advice on the planned data analysis. Many thanks to Professor Roslyn Boyd, Scientific Director of the Queensland Cerebral Palsy and Rehabilitation Research Centre (QCPRRC) for her valuable suggestions regarding methodology and selection of outcome measures and Dr Margaret Mayston (UCL) for her early contribution to the preliminary study design. Most importantly, huge thanks to all the children and their families who have been involved in the development of the research idea, provided advice and input to the study protocol and those who have agreed to participate in the study.

Funding statement

Lesley Katchburian is funded by a National Institute for Health Research (NIHR) Clinical Doctoral Research Fellowship Award [ICA-CDRF-2015-01-037]. The study is also supported by the NIHR GOSH BRC at UCL GOSH Institute of Child Health. The views expressed are those of the authors and not necessarily those of the NIHR or the Department of Health and Social Care.

Conflicts of interest

The authors declare that they have no conflicts of interest in relation to this protocol.

Protocol registration

Full ethical approval for this study (#IRAS 211617) has been granted by London Central Research Ethics Committee (#REC 17/LO/0579). This study is also registered with the joint research and Development office at GOSH and UCL GOSH Institute of Child Health (# 15HN07) and has been adopted into the NIHR Clinical Research Network.

For peer review only

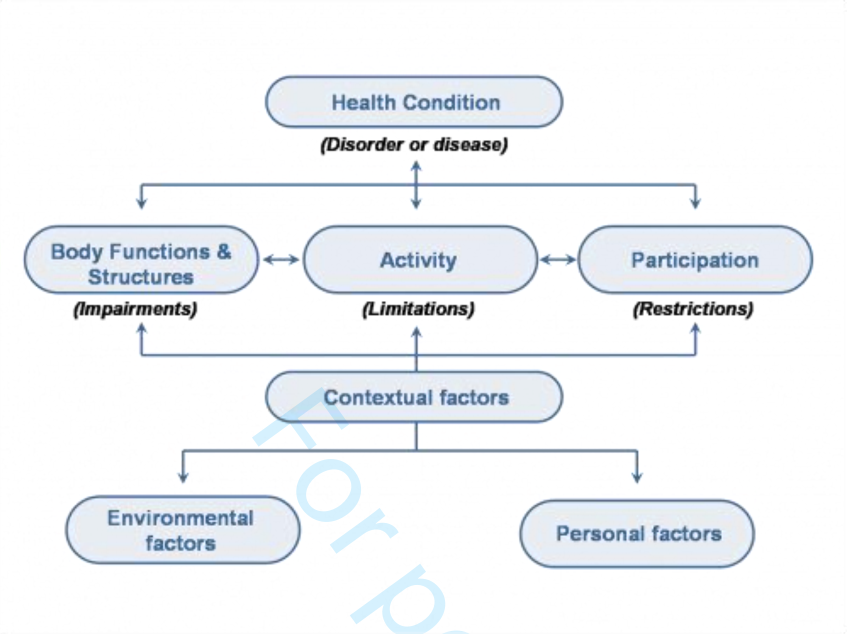

For peer review only

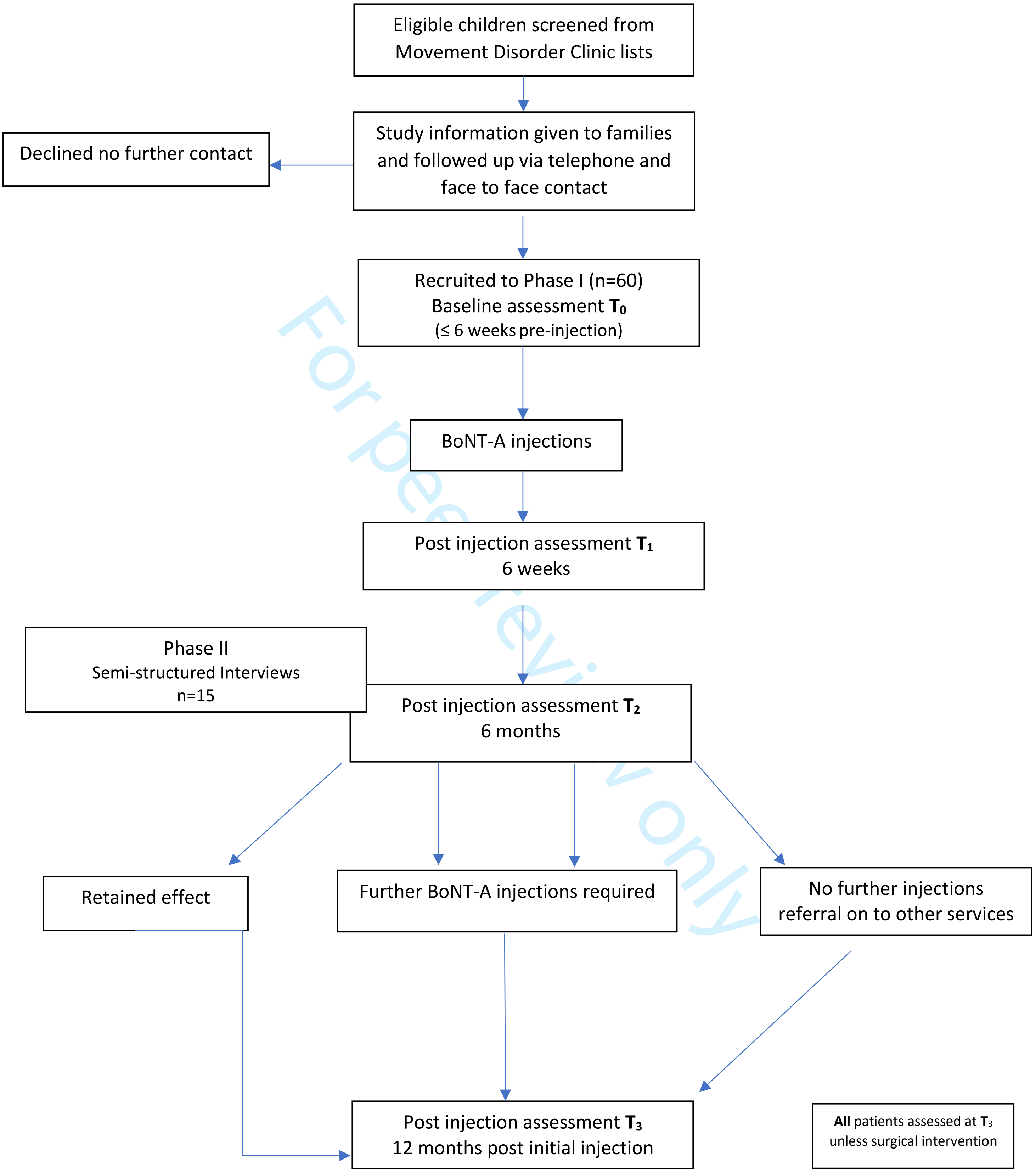

For peer review only

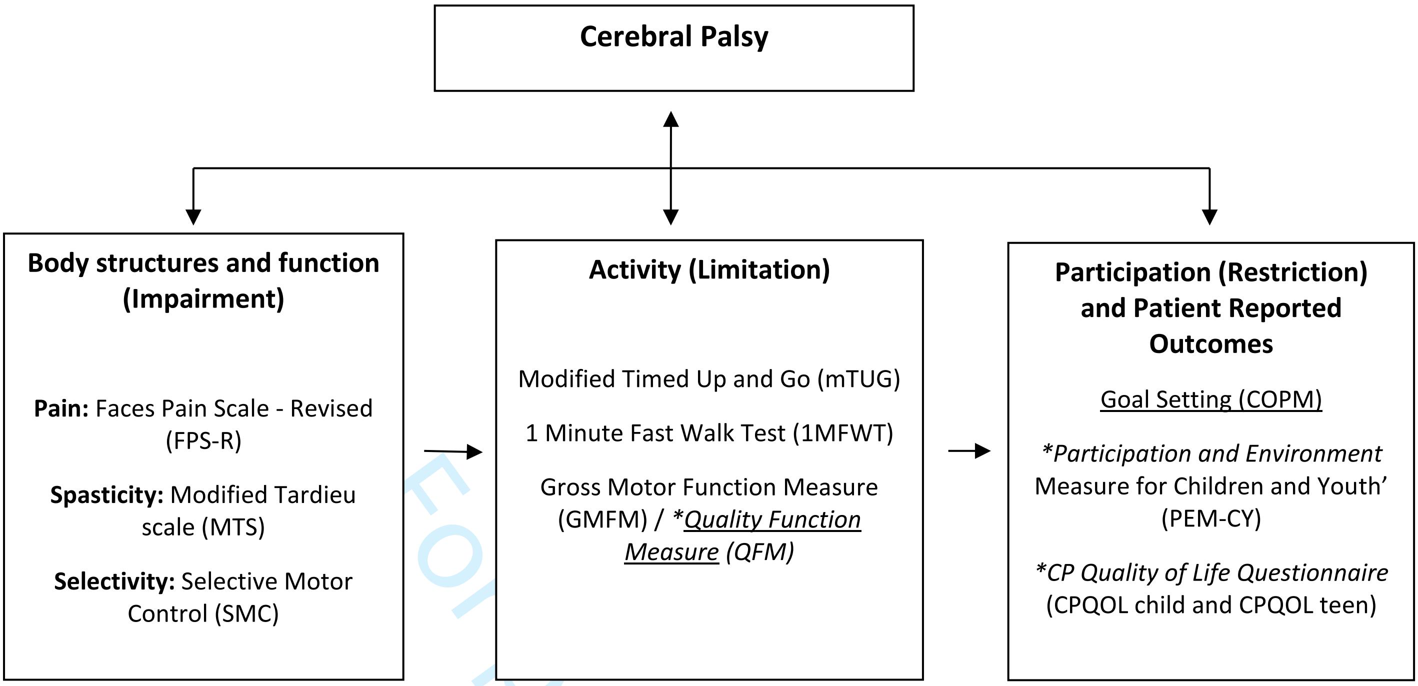

For peer review only

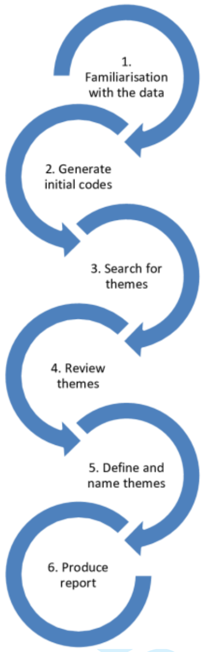
1. Familiarisation with the data
2. Generate initial codes
3. Search for themes
4. Review themes
5. Define and name themes
6. Produce report

BMJ Open

Protocol for The Toxin Study: Understanding clinical and patient reported response of children and young people with cerebral palsy to intramuscular lower limb Botulinum Toxin-A injections, exploring all domains of the ICF: A pragmatic longitudinal observational study using a prospective one group repeated measures design

Journal:	*BMJ Open*
Manuscript ID	bmjopen-2021-049542.R1
Article Type:	Protocol
Date Submitted by the Author:	16-Mar-2021
Complete List of Authors:	Katchburian, Lesley; Great Ormond Street Hospital For Children NHS Foundation Trust, The Wolfson Neurodisability Movement Disorder Service; UCL, GOSH Institute of Child<br>Oulton, Kate; Centre for Outcomes and Experience Research in Children's Health, Illness and Disability (ORCHID)<br>Main, Eleanor; University College London Institute of Child Health, Physiotherapy,<br>Morris, Christopher; University of Exeter Medical School,<br>Carr, Lucinda; Great Ormond Street Hospital for Children, Neuro Science
**Primary Subject Heading**:	Paediatrics
Secondary Subject Heading:	Neurology
Keywords:	Paediatric neurology < NEUROLOGY, Developmental neurology & neurodisability < PAEDIATRICS, REHABILITATION MEDICINE

SCHOLARONE™
Manuscripts

BMJ

I, the Submitting Author has the right to grant and does grant on behalf of all authors of the Work (as defined in the below author licence), an exclusive licence and/or a non-exclusive licence for contributions from authors who are: i) UK Crown employees; ii) where BMJ has agreed a CC-BY licence shall apply, and/or iii) in accordance with the terms applicable for US Federal Government officers or employees acting as part of their official duties; on a worldwide, perpetual, irrevocable, royalty-free basis to BMJ Publishing Group Ltd ("BMJ") its licensees and where the relevant Journal is co-owned by BMJ to the co-owners of the Journal, to publish the Work in this journal and any other BMJ products and to exploit all rights, as set out in our licence.

The Submitting Author accepts and understands that any supply made under these terms is made by BMJ to the Submitting Author unless you are acting as an employee on behalf of your employer or a postgraduate student of an affiliated institution which is paying any applicable article publishing charge ("APC") for Open Access articles. Where the Submitting Author wishes to make the Work available on an Open Access basis (and intends to pay the relevant APC), the terms of reuse of such Open Access shall be governed by a Creative Commons licence – details of these licences and which Creative Commons licence will apply to this Work are set out in our licence referred to above.

Other than as permitted in any relevant BMJ Author's Self Archiving Policies, I confirm this Work has not been accepted for publication elsewhere, is not being considered for publication elsewhere and does not duplicate material already published. I confirm all authors consent to publication of this Work and authorise the granting of this licence.

For peer review only

Protocol for The Toxin Study: Understanding clinical and patient reported response of children and young people with cerebral palsy to intramuscular lower limb Botulinum Toxin-A injections, exploring all domains of the ICF: A pragmatic longitudinal observational study using a prospective one group repeated measures design

Authors: Lesley Katchburian[1,3] lesley.katchburian@gosh.nhs.uk, Kate Oulton[2] kate.oulton@gosh.nhs.uk, Eleanor Main[3] e.main@ucl.ac.uk, Christopher Morris[4] Christopher.Morris@exeter.ac.uk, Lucinda Carr[1] lucinda.carr@gosh.nhs.uk,

Affiliations: [1] The Wolfson Neurodisability Movement Disorder Service, Great Ormond Street Hospital for Children NHS Foundation Trust, London, UK., [2] The Centre for Outcomes and Experience Research in Children's Health, Illness and Disability (ORCHID), Great Ormond Street Hospital for Children NHS Foundation Trust, London, UK., [3] Faculty of Population Health Sciences, University College London Great Ormond Street Institute of Child Health, London, UK. [4] Peninsula Childhood Disability Research Unit (PenCRU), University of Exeter Medical School, Exeter, UK.,

Corresponding Author: Lesley Katchburian, Lesley.katchburian@gosh.nhs.uk. The Wolfson Neurodisability Movement Disorder Service, Great Ormond Street Hospital for Children NHS Foundation Trust, London, UK.

Keywords: Cerebral palsy, Botulinum Toxin-A, hypertonia, ICF, Quality of movement

Word Count: 4229 words

ABSTRACT

Introduction: Botulinum Toxin-A (BoNT-A) is an accepted treatment modality for the management of hypertonia in children and young people with cerebral palsy (CYPwCP). Nevertheless, there are concerns about the long-term effects of BoNT-A, with a lack of consensus regarding the most meaningful outcome measures to guide its use. Most evidence to date is based on short-term outcomes, related to changes at impairment level (restrictions of body functions and structures), rather than changes in adaptive skills (enabling both activity and participation). The proposed study aims to evaluate clinical and patient reported outcomes in ambulant CYPwCP receiving lower limb BoNT-A injections over a 12-month period within all domains of the World Health Organisation's ICF and health-related quality of life (HRQoL).

Methods and Analysis: This pragmatic prospective longitudinal observational study will use a one group repeated measures design. Sixty CYPwCP classified as Gross Motor Classification Levels I-III aged between 4 and 18 years, will be recruited from an established motor disorders service in London, UK. Standardised clinical and patient reported outcome measures within all ICF domains; body structures and function, activity (including quality of movement), goal attainment, participation and HRQoL will be collected pre-injection and at 6 weeks, 6 months and up to 12 months post injection. A representative subgroup of children and carers will participate in a qualitative component of the study, exploring how their experience of BoNT-A treatment relates to clinical outcome measures.

Ethics and Dissemination: Central London Research Ethics Committee has granted ethics approval (#IRAS 211617 #REC 17/LO/0579). Findings will be disseminated in peer-reviewed publications,

conferences, and via networks to participants and relevant stakeholders using a variety of accessible formats including social media.

ARTICLE SUMMARY

Strengths and limitations of this study

1. To our knowledge this is the first published use of the 'Quality Function Measure' (QFM) to objectively evaluate quality of movement (QoM) following BoNT-A treatment and explore whether any change in QoM translates into changes in outcome within activities, participation and health related quality of life.

2. This pragmatic mixed methods observational study will evaluate response to lower limb botulinum A (BoNT-A) injections in ambulant CYPwCP using standardised outcome measures within ***all*** domains of the ICF over a twelve-month period.

3. This study is designed to optimise targeting of BoNT-A treatment by identifying patterns of response to BoNT-A treatment across all ICF domains in order to assist clinicians and families in making informed decisions about future treatment.

4. This is a pragmatic one group study design, conducted from a single tertiary (specialist children's) centre. Participants will continue to receive their routine rehabilitation therapy and orthotic provision at local community level. Individualized intervention parameters may make study replication difficult in other settings.

For peer review only

INTRODUCTION

Cerebral Palsy (CP) is the most common cause of physical disability in childhood (1). Although the initial brain insult is described as static, the effects of the neurological involvement are dynamic and change with time and growth of the child (2) Increased tone (hypertonia) is considered one of the primary motor impairments in children and young people with CP (CYPwCP)(3) and a significant contributor to secondary musculoskeletal impairments impacting on activity and participation(4).

Since the 1990's, intramuscular Botulinum neurotoxin A (BoNT-A) has become an internationally accepted treatment modality for the management of hypertonia in overactive muscle groups (5). BoNT-A once injected into the hypertonic muscle produces a 'reversible' temporary localised muscle weakness by blocking acetylcholine release at the neuromuscular junction. The pharmacological effect is said to last for 12-16 weeks, and the ability of BoNT-A to reduce focal hypertonia in ambulant CYPwCP has been well documented (6-8). Whilst the effects can be observed within 24-72 hours following injection, the period of clinically useful relaxation is reported to last between three to six months (9, 10).

The progression of dynamic contracture to fixed contracture is a fundamental issue in the care of the child with CP. The period of decreased hypertonia following BoNT-A provides a 'window of opportunity' for therapy to address specific predetermined goals of rehabilitation, such as stretching and strengthening of muscles, increased range of motion of joints, improved postural management and pain relief. Despite the temporary effect of injections, gains in motor function have been reported to last as long as twelve months (11).

Several studies have demonstrated the benefits of BoNT-A injections for ambulant CYPwCP in Gross Motor Functional Classification System (GMFCS) Levels I-III (12), particularly at single level use (injection at one level; e.g. the calf complex to treat equinus foot posture) (3, 13-17) . A meta-analysis of double-blind, randomized controlled clinical trials (RCT's) confirmed superiority of BoNT-A over placebo injections into the calf complex on improvement of gait in patients with spastic equinus (18). BoNT-A treatment, in combination with other rehabilitation treatments , has resulted in a significant improvement in functional goal attainment over and above those in a non-BoNT-A treatment group (19), with improvements in gait parameters, pain reduction and splint tolerance (20-23).

Nevertheless, despite positive outcomes for single level use, RCTs of the effectiveness of multi-level use of BoNT-A injections report mixed results. (24, 25). Although a recent systematic review of interventions for CYPwCP, identified BoNT-A treatment as one of few interventions with a sound evidence base (26). A recent Cochrane Collaboration report by Blumetti et al., reviewed 31 studies

For peer review only

assessing 1508 participants and concluded that there is limited evidence to show that compared to placebo or usual care BoNT-A improves walking, joint motion, satisfaction with outcome of treatment and muscle spasticity in CYPwCP (27). Sample sizes in BoNT-A studies are often small and predominately based on short-term outcome (3-6 months) with few assessing outcomes beyond six months.

Although it is widely acknowledged that BoNT-A treatment is not a 'stand-alone' treatment, detailed information regarding the adjunctive measures used in conjunction with BoNT-A is often lacking, making evaluation of its efficacy difficult (28-30). Some authors have highlighted the difficulty in relating changes in impairment measures following BoNT-A treatment to functional improvements in CYPwCP, with little reference made to minimal clinically important differences (MCIDs) (24). This raises concerns that current standardised outcome measures, focusing predominantly on impairment measures (without relating this to other domains of the ICF), may lack the sensitivity to pick up meaningful changes following injections, or indeed overestimate treatment effects if these do not relate to MCIDs, all highly pertinent when planning repeat treatment (24, 27, 31).

Recent studies investigating pathophysiological changes within hypertonic muscle have highlighted potential histological changes following both single and repeated BoNT-A treatment (32). A number of authors have suggested potential harm following repeated BoNT-A use (33-36). However, both positive and negative effects have been reported and a variability in measurement techniques and muscles assessed, makes comparison between studies challenging (37-42). Although BoNT-A has been described as a 'reversible treatment' (43), some authors suggest that BoNT-A exposure in CYPwCP may be associated with impaired muscle growth in the short term (44-47) and potential long term atrophy (41, 45, 48).

The WHO's International Classification of Functioning, Disability and Health (ICF) encourages evaluation of adaptive skills (enabling activities and participation) and health related quality of life (HRQoL) in order to target interventions that are meaningful to CYPwCP and their families (Figure 1). However evidence for BoNT-A treatment remains mostly related to measures of body functions and structures and less evidence pertains to activity and participation (22, 25, 49-51). Few trials have explored improvement in the activity and participation domains or QoL, after BoNT-A injections (24, 52) and qualitative data relating to CYP and caregivers experience is rarely incorporated (53-55).

Figure 1: World Health Organisation's ICF schematic representation of living with a health condition.

Longitudinal changes are not well characterised and evidence of impact on CYPwCP is elusive. The uncertainty that exists around BoNT-A treatment has resulted in a call to extend the period that CYPwCP are followed up after BoNT-A treatment beyond the short term, 12 week period (44), with

For peer review only

an imperative to evaluate interventions with BoNT-A using more sensitive outcome measures that evaluate meaningful aspects of health and quality of life for CYPwCP and families. (35, 44, 56-58)

Concern has been raised that BoNT-A treatment may be overprescribed for CYPwCP (35, 36, 38) if there is little guidance about which patients will benefit, risking potential harm if outcomes are unclear (38, 59-61). Whilst clinical evidence suggests that BoNT-A remains a valuable treatment option (15, 58, 62, 63) , there is a need to optimise it's use by developing clear guidelines and robust treatment algorithms in order to predict which children and young people will benefit from the addition of BoNT-A intervention to their overall management programme and when it is preferable to consider other treatment options.

The Toxin Study

This paper describes the protocol for a pragmatic prospective longitudinal observational study in an established paediatric movement disorder service in London, UK. As BoNT-A treatment is considered best practice care for focal hypertonia management in CP (19, 21, 48) there are practical and ethical concerns regarding the inclusion of a 'no treatment' control group and so a comparator was deemed not ethically appropriate.

The aims of this study are to:

1. Investigate clinical and patient reported outcomes (of body structures and function, quality of movement, activities and participation and health-related QoL) associated with lower limb BoNT-A injections in ambulatory CYPwCP over a twelve-month period.
2. Determine any factors associated with a response to BoNT-A treatment.
3. Explore qualitatively how standardised clinical outcome measures relate to the experiences of CYPwCP following BoNT-A treatment.

METHODS

Study design

This is a mixed methods study comprising of two phases:

Phase 1: To meet objectives 1 and 2 we will use a prospective longitudinal study using a one group repeated measures design with each child acting as their own control.

Phase 2: To meet objective 3 we will conduct interviews with a subgroup of CYPwCP and parent/carers from Phase 1 to elicit their experiences, and views of change following BoNT-A treatment.

For peer review only

Using a convergent mixed methods approach, quantitative data from Phase 1 will be synthesised with qualitative data from phase 2 in order to gain understanding of the impacts of BoNT-A treatment (55, 64).

Study sample and recruitment

Potential study participants will be identified from clinical lists of the Movement Disorder Service at Great Ormond Street Hospital for Children (GOSH). All eligible participants will be invited to take part in Phase 1 and will be enrolled sequentially. A subgroup of CYPwCP and their parents/primary carers will then be invited to participate in Phase 2 following review at six months post injection. Purposive sampling will be used to ensure a representative sample of CYPwCP within each GMFCS level.

Inclusion and exclusion criteria

Participants with a confirmed diagnosis of CP meeting the following criteria will be included:(1) ambulant, functioning at GMFCS level I-III; (2) aged between 4 and 18 years (3) prescribed lower limb BoNT-A injections in their clinical management plan for dynamic hypertonia limiting functional goals or causing pain.

Children will be excluded if they have previously had: (1) orthopaedic surgery to the injected muscle (2) neurosurgery for tone reduction (Selective Dorsal Rhizotomy), (3) lower limb BoNT-A injections in the last 6 months, or currently have (4) unrelated musculoskeletal problems such as recent acute injury or congenital structural abnormality, (5) no access to a block of therapy (defined as a minimum of six weekly sessions) post injections, (6) an inability to complete baseline assessments due to capacity, ability or willingness.

Parents: Every effort will be made to support the inclusion of all families invited to participate in the study (including those where English is not their first language). We will use translators when required to ensure that there is sufficient understanding to complete the measures.

INTERVENTION

Motor assessment

An experienced multidisciplinary team (consisting of a Consultant Paediatrician/ Neurologist and Senior Physiotherapist) will identify muscle groups to be injected. Muscle selection will vary between participants according to presence of dynamic hypertonia (based on clinical assessment with reference to standard definitions (65)), related functional impairment and individual goals of the

For peer review only

CYPwCP. All participants will receive a six-week block of therapy following injections from their local therapy teams, delivered locally in the community as per usual care. Details of dosage including frequency, location and type of therapy (e.g. goal directed therapy, strengthening) together with participation activities will be recorded including any additional intervention such as casting or change in orthotic provision. This will be required in order to describe the content and parameters of usual care in as much detail as possible (66).

Administration of BoNT-A

We will follow standard clinical practice prescription of BoNT-A at GOSH which involves administration of 500 U Dysport® (abobotulinumtoxinA, Dysport® Ipsen Ltd) diluted in 1 ml of normal saline, up to a maximum dose of 30 units /kg/body weight or a total dose up to a maximum of 1000 units per injection session. All CYPwCP will continue to receive BoNT-A injections under ultrasound guidance as a day case, either under sedation with local analgesia, or under general anaesthetic. Adverse events will be recorded and standard reporting of dose per muscle and follow up will be as per current clinical policy.

Training and fidelity

Clinical staff collecting study data are experienced members of the tertiary service with extensive experience of working with CYPwCP (mean 21 years, range 13-33 years). A standardised protocol for measurement and documentation is used in the clinic and an additional study manual with instructions for clinicians will be used to ensure consistency during the study. Two half day training sessions will be provided for clinicians collecting study data prior to the start of recruitment. Monthly meetings with clinicians by the research team will ensure consistency and adherence to study protocol.

Study Procedures & Data Collection

Staggered patient recruitment and data collection will commence in September 2017 and each participant will be followed up for a period of twelve months (**Error! Reference source not found.**). Data collection and final analysis of participant data is expected to be completed by September 2021. All decisions regarding clinical care, assessment frequency and BoNT-A injections will continue as per usual clinical practice. Standardised clinical assessments and outcome measures will be performed at four time points T_0 -T_3. The timings and rationale for these are summarised in Table 1

For peer review only

T_0	**Pre-injection baseline measures**	**1- 6 weeks before injection**
T_1	**6 weeks post injection**	*Estimated time to reach target threshold for BoNT- A* ***'evaluation of efficacy of injections'***
T_2	**6 months following injection**	*Expected completion of pharmacological action -* ***'evaluation of retention of effects post injection'***
	At T_2 **as per usual clinical practice** there are three possible outcomes for participants<br>• Favourable response to injections with retention of effects – no further injections indicated at this time<br>• Favourable response to injections- listed for a second injection cycle<br>• Non favourable response to injections- discharged to other services (e.g. neurosurgery/orthopaedics)	
T_3	**12 months following initial injection**	**End of study**

Table 1: Timings of study assessments T0-T3

All participants *who have not undergone a surgical procedure* will be assessed at T_3, independent of outcome at T_2. This will facilitate analysis of factors associated with changes in impairment, activity, participation and HRQoL following BoNT-A treatment and evaluate time to re-injection over 12 months. . The need for re-injection will be determined by clinical examination (documentation of a technical response e.g. change in MTS), evaluation of goal (COPM) scores and in consultation with families and local team as per usual clinical practice.

Figure 2: Study design flowchart of patient recruitment and data collection during the study

Validated outcome measures for all ICF domains

The standardised outcome measures used in the study are summarised within the ICF domains in Figure 3 with administration details of the measures summarised in Table 2. Outcome measures follow GOSH standard clinical practice with primary outcome measures marked in italics*. Patient assessment takes between 60-90 minutes.

Figure 3: Schematic representation of ICF Domains including standardised outcome measures used in the study.

For peer review only - http://bmjopen.bmj.com/site/about/guidelines.xhtml

Table 2: Summary of outcome measures used in the study

ICF Domains	Assessment	Outcome Measures	Method of Administration	Units	Description
Body functions and structures<br><br>**(impairment)**	Hypertonia and dynamic range of movement	**Modified Tardieu Scale (MTS)**	Standardised goniometry placement	Degrees	**MTS** measured at injected muscles. The difference between the slow stretch R_2 and a fast stretch R_1 is reported as the 'dynamic range' and when a difference is present this 'dynamic range' is reported to be amenable to treatment with BoNT-A.
	Pain	**Faces Pain Scale (FPS-R)**	Score assigned by CYP	Score 0- 10	**FPS-R** which has been shown to have good psychometric properties for pain reporting (modified for use by carer when CYP unable to self-report)
	Selective Motor Control	**Selective Motor Control scale (SMC)**	Score assigned by clinician	Score 0-4	**SMC** Selective motor control of the ankle is assessed using a standardised test procedure
Activity<br>**(functional limitation)**	Gross motor function	**Gross Motor Function Measure (GMFM)**<br>**(D&E walk/run/jump dimensions)**	**Video recorded****<br>Standardised assessment form<br>Scored by clinician	% score	The **GMFM is** designed to evaluate change in gross motor function over time in CYPwCP . It is the standard outcome assessment tool for clinical intervention in CP Dimensions D& E will be used and are considered a **Proxy Functional Gait Measure**<br>*Families are consented for video storage in accordance with the Great Ormond Street Hospital policy*
	Quality of Gross Motor Function	***Quality-Function Measure (QFM)*****	** scored by PI later from GMFM above	% score for 5 quality attributes	The ***QFM*** an observational validated measure captures the quality of movement of the GMFM (D&E) as above post BoNT-A may be more sensitive to change than GMFM alone *scored from video no extra assessment time for CYP*
	Balance/ functional mobility	**Modified Timed up and Go Test (mTUG)**	**Timed standardised test**<br>(from sitting CYP stands and walks distance 3m touches star returns to seat)	Seconds	**mTUG** integrates transitions and walking skills and provides a meaningful measure of capability. It has been shown to be a reliable outcome measure for assessing functional mobility in CYPwCP. **Proxy Functional Gait Measure**
	Walking ability (Efficiency)	**1-minute fast walk test (1MFWT)**	**Distance recorded**<br>(5 minutes rest followed by walking for **1** minute in a 9m corridor at maximum walking speed without running. CYP use normal walking aids and wear orthoses).	Metres	**1MFWT** is a good discriminator of functional ability for dynamic balance, muscle performance and endurance.<br>**Proxy Functional Gait Measure**
Participation<br>**(restriction)**	Involvement in daily activities	**Participation and Environment Measure for Children and Youth' (PEM-CY)**	**Parent- reported Questionnaire**<br>(25 item~ 25 minutes to complete)<br>*Answered at home or in clinic*	Summary Score	The **PEM-CY** assesses participation frequency and involvement in home school and community, along with environmental factors within these settings. Can be completed online at home or in clinic on a handheld device/ paper format whilst waiting for the child's assessment to be completed.
Health Related Quality of life<br>H	Quality of life across seven domains	**Cerebral Palsy Quality of life measure (CPQOL)**	**CYP (or proxy) reported Questionnaire**<br>CP QOL-Child / CP QOL Teen<br>*Answered at home or in clinic*	Mean Domain Score	The ***CPQOL-Child*** is designed to assess condition specific quality of life of children across seven domains for children aged 4-12 years - parent report for children aged 4 to 12 years and a self-report for children aged 9 to 12 years (52-66 items).<br>***CPQOL- Teen*** 13-18 years. Adolescent self-report version and a parent version (72-89 items).
Goal setting<br>**(ICF domains)**	Selection of goals CYP hope to improve following BoNT-A	***Modified Canadian Occupational Performance Measure (mCOPM)***	In clinic 3 Goals set by CYP and family pre injection<br>Scores assigned post injection	Score<br>(1-10)	**mCOPM** rates perception and satisfaction of CYP's performance on a 1–10 ordinal scale. A score change of> two points is considered clinically significant

For peer review only

Classification of the participants

Gross Motor Function Classification System -Expanded and Revised (GMFCS-E&R)

The GMFCS-E&R (12) is an internationally recognised 5 level system to classify the motor abilities of CYPwCP aged 4-18 years, with Level I CYPwCP being identified as the most physically able and Level V the least. It is valid and reliable and frequently used in both research and clinical practice. Only ambulant CYPwCP classified as GMFCS Levels I-III will be included in this study.

Classification of Cerebral Palsy

Participants are classified according to the distribution (unilateral or bilateral) and tone presentation (hypertonia will be identified as predominantly spastic, dystonic or mixed in type) as identified by SCPE (67)

OUTCOME MEASURES

Primary outcome measures

Quality Function Measure QFM[1] is an observational criterion referenced measure designed to evaluate the Quality of Movement (QoM) in standing and walking skills in CYPwCP (68). It is used in conjunction with the Gross Motor Function Measure (GMFM) using Dimensions D and E, which focus on 'standing' and 'walking, jumping and running skills' which are considered proxy clinical gait measures The GMFM is the 'gold standard' tool for evaluating gross motor function in CYPwCP, evaluating 'how much' of a gross motor skill a child can perform (69). However, there are concerns regarding GMFM's sensitivity in capturing subtle yet meaningful change post intervention, due to 'ceiling effects' of the measure when used with ambulant CYPwCP in GMFCS levels I-III (22, 68, 70). The QFM scores movement quality and assesses 'how well' a child performs gross motor tasks (68). It has shown excellent rater and test-retest reliability (ICC 0.89-0.97). Minimal detectable change estimates (6-9%) suggest that the scale has potential as an evaluative measure (V.Wright, personal communication, May 8, 2018). However, to date, there are no published studies evaluating the responsiveness of QFM following BoNT-A injections.

In order to minimise bias, the video analyst will be blinded to the stage of treatment and assessment time point (i.e. pre T_0 or post $T_{1\text{-}3}$ injection). To conceal these time points, each video containing GMFM D & E (standing and walking) dimensions is anonymised and randomly allocated a letter by a co-worker not involved in the service. To minimise recall bias, a time lag will be built in of two weeks and videos of at least 10 other children will be scored before scoring the video of the same child at a

[1] Primary outcome measures for The Toxin Study

For peer review only

different assessment time point. This is in keeping with previous QFM reliability studies which suggest a gap of two weeks before evaluating test-retest scores (68, 70). Data will be entered into a secure data base without access to previous scores until scoring for all assessment time points is complete.

Canadian Occupational Performance Measure (COPM)[1] is a goal attainment tool modified for use in the paediatric population and frequently used in neurodisability research (71-73). It identifies concerns regarding 'occupational performance issues' (OPIs) i.e. the ability to carry out functional tasks allowing the identification of goals and has been used to document change post BoNT-A rehabilitation (74). In the paediatric population areas of concern in a child's self-care, activity and leisure are explored during the pre-assessment appointment with the clinical team. COPM has demonstrated high re-test reliability (ICC 0.76-0.89), sensitivity to change and good content, construct and criterion validity for CYPwCP receiving BoNT-A (75, 76). Families are asked to identify up to three areas of concern which they and their child hope to improve following lower limb BoNT-A injections. Whenever possible, goals are set with the child's input (77). The child and parents are asked to rate their perception of both current performance and their satisfaction with this performance on a 1–10 ordinal scale. A score change of two or more points is considered clinically significant (72, 78).

Secondary outcome measures

Modified Tardieu Scale (MTS) is a recognised clinical measure to differentiate dynamic spasticity from fixed contracture in a muscle. It determines the passive range of movement at two different movement velocities fast (R1) measuring dynamic muscle length and slow (R2) measuring static muscle length, with the relative difference between the two (R2-R1) determining the dynamic tone component of the muscle contracture (79, 80). It is measured in degrees with a universal goniometer using standardised testing positions for each muscle. It has been postulated that the larger the dynamic tone component, the more amenable it is to treatment with BoNT-A (78). It is more effective than the Modified Ashworth Scale (MAS) in identifying the presence of spasticity (88.9%, kappa= 0.73, p=0.000) and the presence of contracture (77.8%, Kappa=0.503, p=008).

Faces Pain scale (FPS-R) is a self-report measure of pain intensity developed for children which has been shown to have good psychometric properties for pain reporting (modified for use by carer when the child is unable to self-report). The scale shows a close linear relationship with visual

For peer review only - http://bmjopen.bmj.com/site/about/guidelines.xhtml

analogue scales. It is measured by an ordinal scale from 0-10 with pictorial representation of faces. A change score of 2 or more is said to be clinically significant (81).

Selective Motor Control (SMC) is a good discriminator of selective motor control and assesses a child's ability to voluntarily and selectively control the dorsiflexors of the ankle (77). It is measured by an ordinal scale between 0 and 4. Higher score indicates better selectivity, inter-rater agreement has been shown to be moderate to good (Kw= 0.58-0.77) with strong test-retest reliability (Kw=0.88-1) (79, 82).

One Minute Fast Walk Test (1MFWT) has been chosen as a proxy gait measure and is a good discriminator of functional ability for dynamic balance, muscle performance and endurance (83). Participants walk for 1 minute along a 9-metre corridor at maximum speed without running. They are able to use their usual walking aids and orthoses. Distance is calculated to the nearest 10cm. It shows concurrent validity with the GMFM, with significant correlation between GMFM score and distance walked (r=0.92) and good reliability (ICC: 0.97)(84).

Modified Timed Up and Go (mTUG) is a good discriminator of balance, anticipatory postural control, and functional mobility in CYPwCP. Participants are timed rising from a chair, walking 3 metres touching a star on the wall and returning to sit down as quickly as they can without running. Participants are able to use their usual walking aids and orthoses. The measure differentiates performance between CYPwCP at GMFCS levels I-III. It has shown excellent inter-rater reliability (ICC 0.83-0.99)(85)

Participation Environment Measure -Child and Youth (PEM-CY) is an innovative parent reported participation measure for use with CYP between 4-18 years. It measures CYPwCP's participation at home (10 items), school (5 items) and community (10 items), taking into account the environmental challenges within each setting. Summary scores for participation frequency, child's involvement and parental desire for change are calculated for all domains. PEM-CY has demonstrated good internal consistency (ICC 0.72-0.83) and test-retest reliability (ICC 0.76-0.89) (86).

Cerebral Palsy Quality of Life Measure (CPQOL) is a HRQoL assessment administered by questionnaire, specifically designed for CYPwCP. It quantifies 'well-being' across seven key HRQoL domains. Items are scored on a nine-point rating scale, then summed and averaged to generate seven domain scores. There are two child reported (CPQOL-Child: 9-12y and CPQOL-Teen:13-18y) and two proxy reported versions dependent on child's age and cognitive ability (CPQOL-Child

For peer review only

Primary care giver 4-12 y and CPQOL-Teen Primary care giver13-18y). The questionnaire designers have stipulated a minimum age of 9 years for child self- reporting, however significant disagreement has been reported between child and parent proxy reports in many HRQoL instruments and the child's perspective will be sought whenever possible in this study (87). The CPQOL has demonstrated good internal consistency (Child ICC 0.74-0.92; Teen 0.81-0.96), and adequate test-retest reliability (Child ICC 0.76-0.89; Teen 0.59-0.83) (88, 89).

Semi-structured interviews

Interviews will be used to elicit CYPwCP and parent/carers views of change following BoNT-A treatment. These are expected to last 60 minutes for parents and up to 30 minutes for children and will take place at home or in the hospital (depending on family preference). Interviews will be guided by a predetermined interview schedule, which will explore perceived change across all ICF domains to facilitate comparison with quantitative outcome measures, as well as investigate the acceptability of the standardised outcome measures used in Part 1 of the study. Parents will be interviewed separately from CYPwCP although individual preference for parents to be present or absent during interviews with their child will be respected. Interviews with CYPwCP will be tailored to their age, cognitive and communication ability using a variety of different techniques, including art-based activities. Offering a toolkit of different creative techniques will ensure that the activities will be accessible to all CYPwCP involved in the interviews, acknowledging not only their different skills and personalities but also their cognitive and physical abilities which is particularly important in this population of CYP (90). To ensure a representative sample of participants for Part 2, CYPwCP with a good, moderate and poor response to toxin (determined by the clinical team at the six-month assessment) within each GMFCS level (I-III) will be invited to take part in Part 2. All interviews will be audio recorded with permission from participants.

For peer review only

Sample size

Phase 1: This study has been powered to detect a difference on one of the two primary outcome measures, The Canadian Occupational Performance Measure (COPM). The sample size power calculation is based on anticipated change in the COPM goal performance at the primary end point T1 (6 weeks post intervention) after BoNT-A treatment. A change in score of two or more points on the performance scale of the COPM would be considered clinically meaningful (72, 78). An earlier study of lower limb BoNT-A intramuscular injections yielded standard deviations between 1.4 and 1.7 for COPM performance (77) . Based on a conservative estimate using a mean change of two points on the COPM performance scale (power 0.8, two tailed, $p<0.05$), 36 participants (12 in each

GMFCS level) are required. Allowing for attrition and missing data over a twelve-month period a total of 60 participants (20 in each GMFCS level) will be recruited for Part I of the study.

As there are no studies reporting Quality Function Measure (QFM) (68) as a primary outcome measure following BoNT-A injections, no data exists to inform a power calculation. It is anticipated that the results from this study will provide power calculation data for use in future multi-centre trials using QFM.

Phase 2: A sample size of approximately 15 CYPwCP (5 from each of the GMFCS levels I-III) and their parent/carer is anticipated to be sufficient to reach thematic saturation for the qualitative element of the study (91)

Data Analysis

Descriptive analysis of the prospective study will provide information on baseline characteristics of impairment, activity, participation and HRQoL. Means and standard deviations will be used for normally distributed data and medians and inter-quartiles ranges for skewed data. Other demographic data will be described in a similar way with frequencies and proportions used for categorical information. The primary outcomes of the intervention will be assessed using generalised estimating equations for longitudinal analysis to evaluate differences in continuous data at post intervention time points. Multilevel regression models will be used to investigate the importance of the various potential predictors over a 12-month period on the 4 main outcomes, impairment, activity, participation and HRQoL Firstly, univariate relationships will be explored and then if appropriate we will fit a multivariable model for each outcome.

All interviews will be transcribed verbatim and transcripts checked by the interviewer. Qualitative data will be analysed using Braun and Clarke's six phase framework for thematic analysis Figure 4 (92). Two researchers (LK,KO) will undertake analysis of the transcripts to determine consistent themes and will then map these onto results from standardised outcomes. This will allow themes to emerge which can then be used to evaluate how closely standardised outcome measures relate to family experience.

Figure 4: Braun and Clarke's Six step approach to thematic analysis

Participant and Data management

Electronic data will be managed through a secure data base held in GOSH. Participants will be allocated an identification code which will be used to de-identify their files and forms. Pseudonyms will be used in all reports and publications and direct quotations will be anonymised. Paper documents and other manual files will be appropriately filed and stored securely in a locked filing

For peer review only

cabinet at GOSH. Demographic information and consent forms will be stored separately to research data. Classification measures, child demographics and related information will be taken prior to baseline for the purposes of stratification and description of the sample. Audio and Visual recordings will be uploaded onto secure password protected encrypted NHS computers and deleted from the recording device immediately after uploading. This protocol has been reviewed and approved by the Research and Development team and Caldecott Guardian at GOSH.

Patient and Public Involvement (PPI)

Children and young people with CP and their parents have been involved in the development of this project, exploring the importance of the research, the appropriateness of the research questions, the acceptability of the research methods and best methods for disseminating findings. Fifteen ambulant CYPwCP receiving BoNT-A treatment at GOSH and their parents were consulted individually. A wider population of CYPwCP and their parents were also consulted via the SCOPE website and the advisory groups for CYPwCP at Brunel University and Young People's Advisory Group (YPAG) at GOSH.

Reference Group/ Steering Group

Three parents (all mothers) and three CYPwCP (two boys and one girl-not participants) continue advising on the study through membership of the study steering group (parents) or reference group (CYP). Three professionals (two physiotherapists and one doctor) are also part of the steering group. By including CYPwCP and families perspectives together with regular contributions from practicing clinicians within the service we hope to ensure the inclusion of important values and preferences from families and clinicians alike, ensuring that the findings of the study remain relevant and applicable to the management of ambulant CYPwCP (24, 93, 94)

DISCUSSION

This paper presents the protocol for a novel pragmatic prospective longitudinal observational study of BoNT-A use in ambulant CYPwCP at an established tertiary children's centre in the UK. It will measure outcomes across all domains of the ICF and HRQoL over a 12-month period, to observe change and examine factors associated with positive or negative response to lower limb BoNT-A injections. The introduction of a standardised outcome measure, QFM, to evaluate any change in movement quality following BoNT-A treatment will further inform exploration of the relationship between tone reduction and changes in activity, as well as any influence on participation and HRQoL for our cohort of CYPwCP. . Mixed methods research designs are considered by many to be essential

For peer review only

in the study of therapeutic interventions in CP (55). Including qualitative study data will ensure that the experiences of CYPwCP following BoNT-A injections are elucidated, considered and further understood.

This study, by identifying patterns of response to BoNT-A injections in key aspects of health across all the ICF domains, will provide clinicians and families with meaningful information to inform future treatment planning and optimise the use of BoNT-A in CYPwCP. Evidence will be generated about the appropriateness of the outcome measures used in detecting meaningful change after BoNT-A injections as well as their acceptability to CYP. In addition, the relationship between the standardised outcome measures used to capture treatment effect and the perception of outcome by children and their families will be investigated.

The results of this study will assist in the development of a pragmatic set of standardised clinical outcome measures (recognising minimum clinically important differences) in order to evaluate the effects of BoNT-A treatment in this cohort of patients. We hope to inform future evaluative research; working towards closer consensus on patient selection, injection frequency and consideration of how long treatment should continue, in order to assess the value of long-term use of BoNT-A treatment and its role in the management of CYPwCP.

A dissemination strategy has been devised to ensure the findings of this research are made widely accessible to families and professionals in order to maximise impact on the care of CYPwCP. Working in partnership with parent groups will strengthen our dissemination strategy, ensuring findings are shared in a variety of accessible formats reaching a wide range of families, professionals, as well as academics and policy makers helping ensure the findings are translated into practice. We will disseminate the results of the study through international peer reviewed journals and at national and international conferences. A social media strategy will also be developed to ensure dissemination of a plain language summary of findings.

For peer review only

References

1. Odding E, Roebroeck ME, Stam HJ. The epidemiology of cerebral palsy: incidence, impairments and risk factors. Disabil Rehabil. 2006;28(4):183-91.
2. Molenaers G, Van Campenhout A, Fagard K, et al. The use of botulinum toxin A in children with cerebral palsy, with a focus on the lower limb. J Child Orthop. 2010;4(3):183-95.
3. Koman LA, Mooney JF, 3rd, Smith BP, et al. Management of spasticity in cerebral palsy with botulinum-A toxin: report of a preliminary, randomized, double-blind trial. Journal of Pediatric Orthopedics. 1994;14(3):299-303.
4. Koman LA, Paterson Smith B, Balkrishnan R. Spasticity associated with cerebral palsy in children: guidelines for the use of botulinum A toxin. Paediatric Drugs. 2003;5(1):11-23.
5. Ward AB, Molenaers G, Colosimo C, et al. Clinical value of botulinum toxin in neurological indications. European Journal of Neurology. 2006;13 Suppl 4:20-6.
6. Tedroff K, Granath F, Forssberg H, et al. Long-term effects of botulinum toxin A in children with cerebral palsy. Developmental Medicine & Child Neurology. 2009;51(2):120-7.
7. Hoare BJ, Imms C. Upper-limb injection of botulinum toxin-A in children with cerebral palsy: a critical review of the literature and clinical implications for occupational therapists. American Journal of Occupational Therapy. 2004;58(4):389-97.
8. Lukban MB, Rosales RL, Dressler D. Effectiveness of botulinum toxin A for upper and lower limb spasticity in children with cerebral palsy: A summary of evidence. Journal of Neural Transmission. 2009;116(3):319-31.
9. Ryll U, Bastiaenen C, Bie R, et al. Effects of leg muscle botulinum toxin A injections on walking in children with spasticity-related cerebral palsy: a systematic review (Provisional abstract). Developmental Medicine and Child Neurology. 2011;53(3):210-6.
10. Aoki KR, Ranoux D, Wissel J. Using translational medicine to understand clinical differences between botulinum toxin formulations. Eur J Neurol. 2006;13 Suppl 4:10-9.
11. Eliege de Souza MNACD, Renata Calhes Franco MFMS, Fabiano Politti CSO. Effect of Botulinum Toxin a on Spasticity and Function in Children with Cerebral Palsy: A Systematic Review. Med Sci Tech, 2014; 55: 11-15. 2014.
12. Palisano R, Rosenbaum P, Walter S, et al. Development and reliability of a system to classify gross motor function in children with cerebral palsy. Dev Med Child Neurol. 1997;39(4):214-23.
13. Desloovere K. A randomized study of combined botulinum toxin type A and casting in the ambulant child with cerebral palsy using objective outcome measures. European Journal of Neurology. 2001;8(SUPPL. 5):75-87.
14. Gough M, Fairhurst C, Shortland AP. Botulinum toxin and cerebral palsy: time for reflection? Developmental Medicine & Child Neurology. 2005;47(10):709-12.
15. Svehlik M, Kraus T, Steinwender G, et al. Repeated multilevel botulinum toxin A treatment maintains long-term walking ability in children with cerebral palsy. Ceska a Slovenska Neurologie a Neurochirurgie. 2012;75(6):737-41.
16. Delgado MR. The use of botulinum toxin type A in children with cerebral palsy: a retrospective study. European Journal of Neurology. 1999;6:S11-S8.
17. Delgado M, Tilton A, Gormley M, et al. Abobotulinumtoxina injection in muscles outside the gastrocnemius-soleus complex in pediatric patients with lower limb spasticity. Developmental Medicine and Child Neurology. 2017;59 (Supplement 3):80-1.
18. Cardoso ES, Rodrigues BM, Barroso M, et al. Botulinum toxin type A for the treatment of the spastic equinus foot in cerebral palsy. Pediatr Neurol. 2006;34(2):106-9.
19. Wallen M, O'Flaherty SJ, Waugh MCA. Functional outcomes of intramuscular botulinum toxin type A and occupational therapy in the upper limbs of children with cerebral palsy: A randomized controlled trial. Archives of Physical Medicine and Rehabilitation. 2007;88(1):1-10.
20. Heinen F, Desloovere K, Schroeder AS, et al. The updated European Consensus 2009 on the use of Botulinum toxin for children with cerebral palsy. European Journal of Paediatric Neurology. 2010;14(1):45-66.

21. Fehlings D, Novak I, Berweck S, et al. Botulinum toxin assessment, intervention and follow-up for paediatric upper limb hypertonicity: International consensus statement. European Journal of Neurology. 2010;17(SUPPL. 2):38-56.
22. Love SC, Novak I, Kentish M, et al. Botulinum toxin assessment, intervention and after-care for lower limb spasticity in children with cerebral palsy: International consensus statement. European Journal of Neurology. 2010;17(SUPPL. 2):9-37.
23. Metaxiotis D, Siebel A, Doederlein L. Repeated botulinum toxin A injections in the treatment of spastic equinus foot. Clinical Orthopaedics & Related Research. 2002(394):177-85.
24. Wright FV, Rosenbaum PL, Goldsmith CH, et al. How do changes in body functions and structures, activity, and participation relate in children with cerebral palsy? Developmental Medicine & Child Neurology. 2008;50(4):283-9.
25. Strobl W, Theologis T, Brunner R, et al. Best clinical practice in botulinum toxin treatment for children with cerebral palsy. Toxins. 2015;7(5):1629-48.
26. Novak I, McIntyre S, Morgan C, et al. A systematic review of interventions for children with cerebral palsy: state of the evidence. Developmental Medicine & Child Neurology. 2013;55(10):885-910.
27. Blumetti FC, Belloti JC, Tamaoki MJS, et al. Botulinum toxin type A in the treatment of lower limb spasticity in children with cerebral palsy. Cochrane Database of Systematic Reviews. 2019(10).
28. Williams S, Elliott C, Gubbay A, et al. The immediate effect of Botulinum Toxin Type-A on muscle morphology and strength in children with cerebral palsy. Developmental Medicine and Child Neurology. 2012;54:35-6.
29. Schasfoort FC, Pangalila RF, Dallmeijer AJ, et al. Evaluation of the added value of botulinum toxin treatment and intensive physiotherapy to improve body function and structure in children with spastic cerebral palsy. Developmental Medicine and Child Neurology. 2017;59 (Supplement 2):21.
30. Mathevon L, Bonan I, Barnais JL, et al. Adjunct therapies to improve outcomes after botulinum toxin injection in children: A systematic review. Ann Phys Rehabil Med. 2019;62(4):283-90.
31. Williams SA, Reid S, Valentine J, et al. Activity, participation and quality of life in children with cerebral palsy: Can a combined approach of strength training and botulinum neurotoxin type-A help? Developmental Medicine and Child Neurology. 2014;56:76.
32. Booth CM, Cortina-Borja MJ, Theologis TN. Collagen accumulation in muscles of children with cerebral palsy and correlation with severity of spasticity. Dev Med Child Neurol. 2001;43(5):314-20.
33. Hastings-Ison T, Thomason P, Sangeux M, et al. Botox injections for spastic equinus in ambulant children with cerebral palsy: A prospective 3D kinematic study. Developmental Medicine and Child Neurology. 2018;60 (Supplement 1):56-7.
34. Copeland L, Edwards P, Thorley M, et al. Botulinum toxin A for nonambulatory children with cerebral palsy: a double blind randomized controlled trial. The Journal of pediatrics. 2014;165(1):140-6.e4.
35. Multani I, Manji J, Hastings-Ison T, et al. Botulinum Toxin in the Management of Children with Cerebral Palsy. Paediatr Drugs. 2019;21(4):261-81.
36. Gough M. Does botulinum toxin prevent or promote deformity in children with cerebral palsy? Developmental Medicine & Child Neurology. 2009;51(2):89-90.
37. Tedroff K. Short and long term effects of Botulinum toxin A in children. Developmental Medicine and Child Neurology. 2009;51:10.
38. Sim E, Park E, Jung S. Architectural change of gastrocnemius muscle after botulinum toxin injection in children with cerebral palsy. Developmental Medicine and Child Neurology. 2012;54:20-1.
39. Legerlotz K, Matthews KG, McMahon CD, et al. Botulinum toxin-induced paralysis leads to slower myosin heavy chain isoform composition and reduced titin content in juvenile rat gastrocnemius muscle. Muscle & Nerve. 2009;39(4):472-9.

For peer review only

40. Eek MN, Pahlman M, Himmelmann K, et al. Muscle strength after botulinum neurotoxin injection in children with cerebral palsy. Developmental Medicine and Child Neurology. 2014;56:61-2.
41. Fortuna R, Horisberger M, Vaz MA, et al. Do skeletal muscle properties recover following repeat onabotulinum toxin A injections? Journal of Biomechanics. 2013;46(14):2426-33.
42. Minamoto VB, Suzuki KP, Bremner SN, et al. Dramatic changes in muscle contractile and structural properties after 2 botulinum toxin injections. Muscle & Nerve. 2015;52(4):649-57.
43. Ney JP, Joseph KR. Neurologic uses of botulinum neurotoxin type A. Neuropsychiatric disease and treatment. 2007;3(6):785-98.
44. Alexander C, Elliott C, Valentine J, et al. Muscle volume alterations after first botulinum neurotoxin A treatment in children with cerebral palsy: a 6-month prospective cohort study. Developmental Medicine & Child Neurology. 2018;60(11):1165-71.
45. Van Campenhout A, Verhaegen A, Pans S, et al. Botulinum toxin type A injections in the psoas muscle of children with cerebral palsy: Muscle atrophy after motor end plate-targeted injections. Research in Developmental Disabilities. 2013;34(3):1052-8.
46. Park ES, Sim E, Rha DW, et al. Architectural changes of the gastrocnemius muscle after botulinum toxin type A injection in children with cerebral palsy. Yonsei Medical Journal. 2014;55(5):1406-12.
47. Williams SA, Reid S, Elliott C, et al. Muscle volume alterations in spastic muscles immediately following botulinum toxin type-A treatment in children with cerebral palsy. Developmental Medicine and Child Neurology. 2013;55(9):813-20.
48. Barber L, Hastings-Ison T, Baker R, et al. The effects of botulinum toxin injection frequency on calf muscle growth in young children with spastic cerebral palsy: a 12-month prospective study. Journal of children's orthopaedics. 2013;7(5):425-33.
49. NICE. Spasticity in children and young people with non progressive brain disorders. Clinical Guideline 145; . London: National Institute for Health and Clinical Excellence. ; 2012.
50. Anne Whelan M, Delgado Frcpc Faan MR. Practice parameter: pharmacologic treatment of spasticity in children and adolescents with cerebral palsy (an evidence-based review): report of the quality standards subcommittee of the american academy of neurology and the practice committee of the child neurology society...Neurology. 2010 Jan 26;74(4):336-43. Neurology. 2010;75(7):669-.
51. Carr LJ, Cosgrove AP, Gringras P, et al. Position paper on the use of botulinum toxin in cerebral palsy. UK Botulinum Toxin and Cerebral Palsy Working Party. Archives of Disease in Childhood. 1998;79(3):271-3.
52. Read FA, Boyd RN, Barber LA. Longitudinal assessment of gait quality in children with bilateral cerebral palsy following repeated lower limb intramuscular Botulinum toxin-A injections. Research in Developmental Disabilities. 2017;68:35-41.
53. Lowing K, Thews K, Haglund-Akerlind Y, et al. Effects of Botulinum Toxin-A and Goal-Directed Physiotherapy in Children with Cerebral Palsy GMFCS Levels I & II. Physical & Occupational Therapy in Pediatrics. 2017;37(3):268-82.
54. Tilton AH. Evidence-based review of safety and efficacy of botulinum neurotoxin in cerebral palsy. Toxicon. 2015;93:S59.
55. Rosenbaum P. How do we know if interventions in developmental disability are effective? Developmental Medicine & Child Neurology. 2020;62(12):1344-.
56. Nguyen L, Mesterman R, Gorter JW. Development of an inventory of goals using the International Classification of Functioning, Disability and Health in a population of non-ambulatory children and adolescents with cerebral palsy treated with botulinum toxin A. BMC Pediatrics. 2018;18(1):1.
57. Hastings-Ison T, Blackburn C, Graham HK, et al. Optimum frequency of botulinum toxin injections to the gastrocsoleus in children with cerebral palsy-a randomised controlled trial. Developmental Medicine and Child Neurology. 2014;56:27.

For peer review only

58. Langdon K, Copeland L, Edwards P, et al. Comment on: "Botulinum Toxin in the Management of Children with Cerebral Palsy". Pediatric Drugs. 2019.
59. Hastings-Ison T, Sangeux M, Graham H. Botulinum Toxin A injections for spastic equinus in ambulant children with cerebral palsy: Longterm follow-up and time to surgery. Developmental Medicine and Child Neurology. 2017;59 (Supplement 3):33-4.
60. Multani I, Manji J, Tang MJ, et al. Sarcopenia, Cerebral Palsy, and Botulinum Toxin Type A. JBJS reviews. 2019;7(8):e4.
61. Montastruc J, Marque P, Moulis F, et al. Adverse drug reactions of botulinum neurotoxin type A in children with cerebral palsy: a pharmaco-epidemiological study in VigiBase. Dev Med Child Neurol. 2017;59(3):329-34.
62. Delgado M, Tilton A, Gormley M, et al. Dysport (abobotulinumtoxinA) injection in muscles in pediatric patients with lower limb spasticity. Neurology Conference: 69th American Academy of Neurology Annual Meeting, AAN. 2017;88(16 Supplement 1).
63. Dursun N, Bonikowski M, Dabrowski E, et al. Efficacy of Repeat AbobotulinumtoxinA (Dysport®) Injections in Improving Gait in Children with Spastic Cerebral Palsy. Developmental Neurorehabilitation. 2019:1-7.
64. Fetters MD, Curry LA, Creswell JW. Achieving integration in mixed methods designs-principles and practices. Health Serv Res. 2013;48(6 Pt 2):2134-56.
65. Graham HK, Rosenbaum P, Paneth N, et al. Cerebral palsy. Nature Reviews Disease Primers. 2016;2(1):15082.
66. Hoffmann TC, Glasziou PP, Boutron I, et al. Better reporting of interventions: template for intervention description and replication (TIDieR) checklist and guide. BMJ : British Medical Journal. 2014;348:g1687.
67. Gainsborough M, Surman G, Maestri G, et al. Validity and reliability of the guidelines of the surveillance of cerebral palsy in Europe for the classification of cerebral palsy. Dev Med Child Neurol. 2008;50(11):828-31.
68. Wright FV, Rosenbaum P, Fehlings D, et al. The Quality Function Measure: reliability and discriminant validity of a new measure of quality of gross motor movement in ambulatory children with cerebral palsy. Dev Med Child Neurol. 2014;56(8):770-8.
69. Boyce W, Gowland C, Rosenbaum P, et al. The Gross Motor Performance Measure: Validity and Responsiveness of a Measure of Quality of Movement1995. 603-13 p.
70. Tustin K, Gimeno H, Morton E, et al. Rater reliability and scoring duration of the Quality Function Measure in ambulant children with hyperkinetic movement disorders. Dev Med Child Neurol. 2016;58(8):822-8.
71. Law M, Baptiste S, Mills J. Client-centred practice: what does it mean and does it make a difference? Can J Occup Ther. 1995;62(5):250-7.
72. McColl MA, Law M, Baptiste S, et al. Targeted Applications of the Canadian Occupational Performance Measure. Canadian Journal of Occupational Therapy. 2005;72(5):298-300.
73. Kang M, Smith E, Goldsmith CH, et al. Documenting change with the Canadian Occupational Performance Measure for children with cerebral palsy. Developmental Medicine and Child Neurology. 2020;62(10):1154-60.
74. O'Neil ME, Fragala MA, Dumas HM. Physical therapy intervention for children with cerebral palsy who receive botulinum toxin a injections. Pediatr Phys Ther. 2003;15(4):204-15.
75. Fragala MA, O'Neil M E, Russo KJ, et al. Impairment, disability, and satisfaction outcomes after lower-extremity botulinum toxin a injections for children with cerebral palsy. Pediatr Phys Ther. 2002;14(3):132-44.
76. Cusick A, McIntyre S, Novak I, et al. A comparison of goal attainment scaling and the Canadian Occupational Performance Measure for paediatric rehabilitation research. Pediatr Rehabil. 2006;9(2):149-57.
77. Bjornson K, Hays R, Graubert C, et al. Botulinum toxin for spasticity in children with cerebral palsy: a comprehensive evaluation. Pediatrics. 2007;120(1):49-58.

For peer review only

78. Law M, Baptiste S, Carswell A, et al. Canadian Occupational Performance Measure. Ottowa, Ontario: COAT publications; 1998 1998//.
79. Boyd RN, Graham HK. Objective measurement of clinical findings in the use of botulinum toxin type A for the management of children with cerebral palsy. European Journal of Neurology. 1999;6:S23-S35.
80. Love SC, Valentine JP, Blair EM, et al. The effect of botulinum toxin type A on the functional ability of the child with spastic hemiplegia a randomized controlled trial. European Journal of Neurology. 2001;8 Suppl 5:50-8.
81. Hicks CL, von Baeyer CL, Spafford PA, et al. The Faces Pain Scale-Revised: toward a common metric in pediatric pain measurement. Pain. 2001;93(2):173-83.
82. Lowing K, Bexelius A, Carlberg EB. Goal-directed functional therapy: A longitudinal study on gross motor function in children with cerebral palsy. Disability and Rehabilitation. 2010;32(11):908-16.
83. McDowell BC, Kerr C, Parkes J, et al. Validity of a 1 minute walk test for children with cerebral palsy. Dev Med Child Neurol. 2005;47.
84. Scholtes VA, Becher JG, Janssen-Potten YJ, et al. Effectiveness of functional progressive resistance exercise training on walking ability in children with cerebral palsy: a randomized controlled trial. Res Dev Disabil. 2012;33.
85. Carey H, Martin K, Combs-Miller S, et al. Reliability and Responsiveness of the Timed Up and Go Test in Children With Cerebral Palsy. Pediatric Physical Therapy. 2016;28(4):401-8.
86. Coster W, Bedell G, Law M, et al. Psychometric evaluation of the Participation and Environment Measure for Children and Youth. Dev Med Child Neurol. 2011;53(11):1030-7.
87. Upton P, Lawford J, Eiser C. Parent–child agreement across child health-related quality of life instruments: a review of the literature. Quality of Life Research. 2008;17(6):895.
88. Davis E, Mackinnon A, Davern M, et al. Description and psychometric properties of the CP QOL-Teen: a quality of life questionnaire for adolescents with cerebral palsy. Res Dev Disabil. 2013;34.
89. Waters E, Davis E, Mackinnon A, et al. Psychometric properties of the quality of life questionnaire for children with CP. Developmental Medicine & Child Neurology. 2007;49(1):49-55.
90. O'Grady MG, Dusing SC. Reliability and Validity of Play-Based Assessments of Motor and Cognitive Skills for Infants and Young Children: A Systematic Review. Physical Therapy. 2015;95(1):25-38.
91. Fugard AJB, Potts HWW. Supporting thinking on sample sizes for thematic analyses: a quantitative tool. International Journal of Social Research Methodology. 2015;18(6):669-84.
92. Braun V, Clarke V. Using thematic analysis in psychology. Qualitative Research in Psychology. 2006;3(2):77-101.
93. INVOLVE. Involving children and young people in research: Top tips and essential key issues for researchers. INVOLVE, Eastleigh 2016.
94. Nguyen L, Di Rezze B, Mesterman R, et al. Effects of Botulinum Toxin Treatment in Nonambulatory Children and Adolescents With Cerebral Palsy: Understanding Parents' Perspectives. Journal of Child Neurology. 2018;33(11):724-33.

Figure Legends

Figure 1: World Health Organisation's ICF schematic representation of living with a health condition 4
Figure 2: Study design flowchart of patient recruitment and data collection during the study 8
Figure 3: Schematic representation of ICF Domains including standardised outcome measures used in the study 8

Figure 4: Braun and Clarke's Six step approach to thematic analysis..14

Authors contributions

LK, KO, LC, EM, CM conceived and designed the study. LK developed the protocol with input from all authors. LK and LC are responsible for recruiting and supporting all the participants. LK, EM, KO will conduct the data analyses. LK and LC wrote the first draft of the manuscript, all authors reviewed this and have approved the final manuscript.

Acknowledgements

With thanks to the clinical team at The Wolfson Neurodisability Movement Disorder Service at GOSH, especially, Dr. Belinda Crowe, Xanthe Hodgson, Josephine Scerri, Emilie Hupin, Katharine Phillipps, Tessa Burnett, Sarah Foster, Agnieszka Rozborska, Stephanie Cawker. Also Dr Jo Wray from the Centre for Outcomes and Experience Research in Children's Health, Illness and Disability (ORCHID) at GOSH for support and advice on the planned data analysis. Many thanks to Professor Roslyn Boyd, Scientific Director of the Queensland Cerebral Palsy and Rehabilitation Research Centre (QCPRRC) for her valuable suggestions regarding methodology and selection of outcome measures and Dr Margaret Mayston (UCL) for her early contribution to the preliminary study design. Most importantly, huge thanks to all the children and their families who have been involved in the development of the research idea, provided advice and input to the study protocol and those who have agreed to participate in the study.

Funding statement

Lesley Katchburian is funded by a National Institute for Health Research (NIHR) Clinical Doctoral Research Fellowship Award [ICA-CDRF-2015-01-037]. The study is also supported by the NIHR GOSH BRC at UCL GOSH Institute of Child Health. The views expressed are those of the authors and not necessarily those of the NIHR or the Department of Health and Social Care.

Conflicts of interest

The authors declare that they have no conflicts of interest in relation to this protocol.

Protocol registration

Full ethical approval for this study (#IRAS 211617) has been granted by London Central Research Ethics Committee (#REC 17/LO/0579). This study is also registered with the joint research and Development office at GOSH and UCL GOSH Institute of Child Health (# 15HN07) and has been adopted into the NIHR Clinical Research Network.

For peer review only

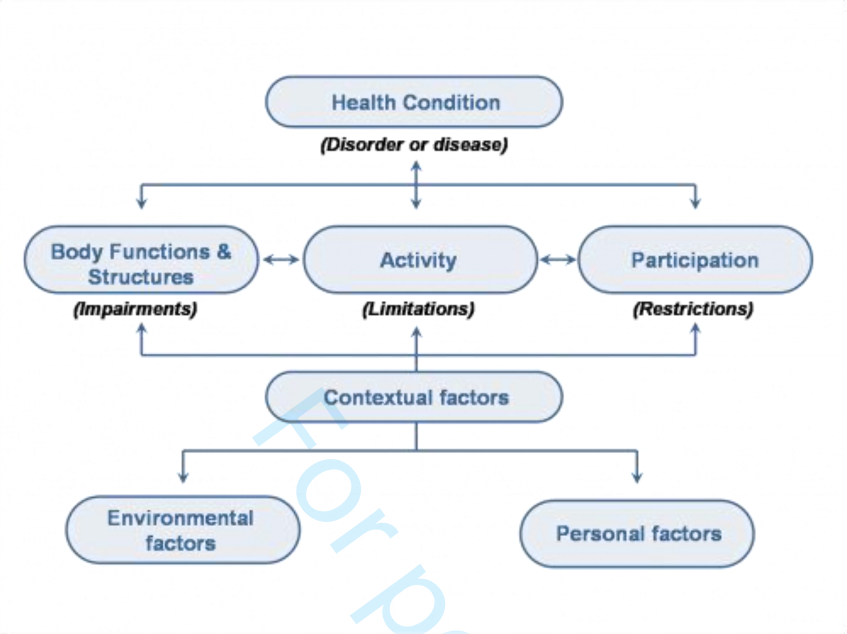

For peer review only

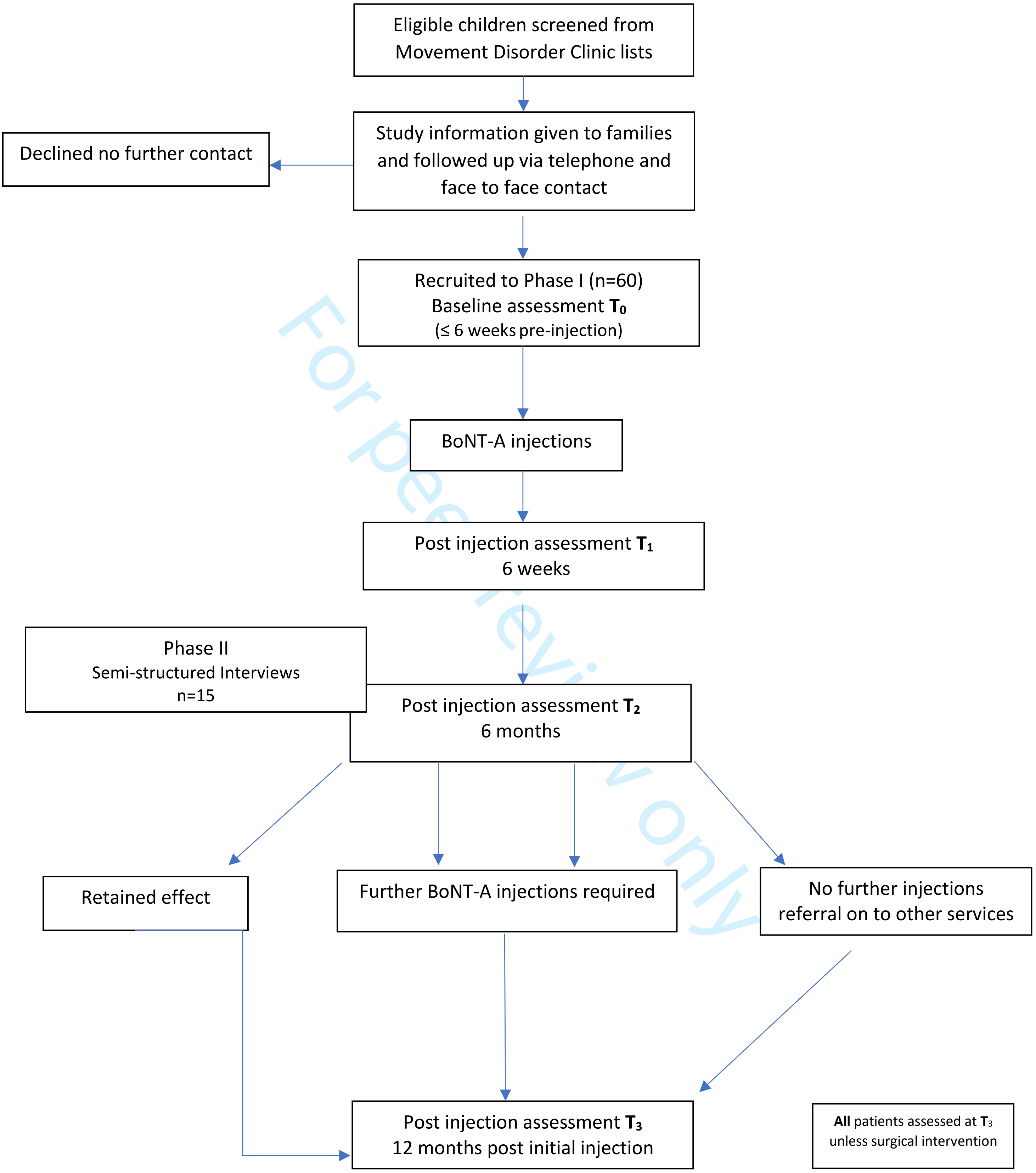

For peer review only

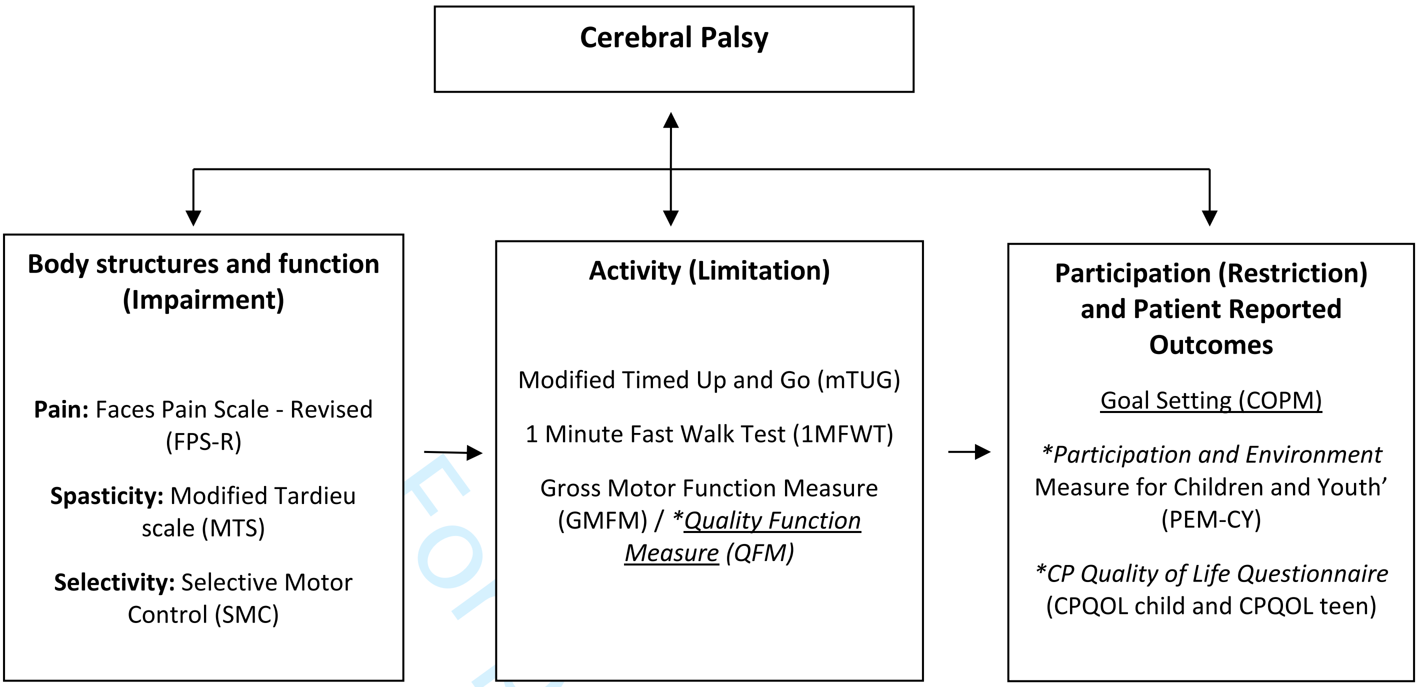

For peer review only

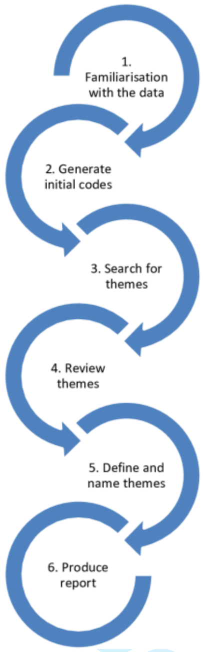
1. Familiarisation with the data
2. Generate initial codes
3. Search for themes
4. Review themes
5. Define and name themes
6. Produce report
